# The Ser/Thr protein kinase FonKin4-poly(ADP-ribose) polymerase FonPARP1 phosphorylation cascade is required for the pathogenicity of watermelon fusarium wilt fungus *Fusarium oxysporum* f. sp. *niveum*

**DOI:** 10.3389/fmicb.2024.1397688

**Published:** 2024-04-16

**Authors:** Jiajing Wang, Yizhou Gao, Xiaohui Xiong, Yuqing Yan, Jiajun Lou, Muhammad Noman, Dayong Li, Fengming Song

**Affiliations:** ^1^Zhejiang Provincial Key Laboratory of Biology of Crop Pathogens and Insects, Institute of Biotechnology, Zhejiang University, Hangzhou, China; ^2^Key Laboratory of Crop Diseases and Insect Pests of Ministry of Agriculture and Rural Affairs, Institute of Biotechnology, Zhejiang University, Hangzhou, China; ^3^State Key Laboratory for Managing Biotic and Chemical Treats to the Quality and Safety of Agro-Products, Institute of Plant Protection and Microbiology, Zhejiang Academy of Agricultural Sciences, Hangzhou, China; ^4^State Key Laboratory of Rice Biology and Breeding, Institute of Biotechnology, Zhejiang University, Hangzhou, China

**Keywords:** watermelon, fusarium wilt (*Fusarium oxysporum* f. sp. *niveum*), PARylation, FonPARP1, FonKin4, pathogenicity

## Abstract

Poly(ADP-ribosyl)ation (PARylation), catalyzed by poly(ADP-ribose) polymerases (PARPs) and hydrolyzed by poly(ADP-ribose) glycohydrolase (PARG), is a kind of post-translational protein modification that is involved in various cellular processes in fungi, plants, and mammals. However, the function of PARPs in plant pathogenic fungi remains unknown. The present study investigated the roles and mechanisms of FonPARP1 in watermelon Fusarium wilt fungus *Fusarium oxysporum* f. sp. *niveum* (*Fon*). *Fon* has a single PARP FonPARP1 and one PARG FonPARG1. FonPARP1 is an active PARP and contributes to *Fon* pathogenicity through regulating its invasive growth within watermelon plants, while FonPARG1 is not required for *Fon* pathogenicity. A serine/threonine protein kinase, FonKin4, was identified as a FonPARP1-interacting partner by LC–MS/MS. FonKin4 is required for vegetative growth, conidiation, macroconidia morphology, abiotic stress response and pathogenicity of *Fon*. The S_TKc domain is sufficient for both enzyme activity and pathogenicity function of FonKin4 in *Fon*. FonKin4 phosphorylates FonPARP1 *in vitro* to enhance its poly(ADP-ribose) polymerase activity; however, FonPARP1 does not PARylate FonKin4. These results establish the FonKin4-FonPARP1 phosphorylation cascade that positively contributes to *Fon* pathogenicity. The present study highlights the importance of PARP-catalyzed protein PARylation in regulating the pathogenicity of *Fon* and other plant pathogenic fungi.

## 1 Introduction

The *Fusarium oxysporum* species complex (FOSC), ranked fifth among the top 10 plant pathogens (Dean et al., 2012), causes devastating vascular wilt diseases in over 150 economically important crops, including tomato, cotton, banana, cucumber, melon, and watermelon, leading to significant global yield losses (Di Pietro et al., 2003; Michielse and Rep, 2009; Gordon, 2017; Husaini et al., 2018). FOSC comprises over 100 formae speciales (f. sp.), each displaying distinct pathogenicity toward various host plant species (Gordon, 2017). These strains can be categorized as either plant pathogenic or nonpathogenic, displaying morphological variations (Edel-Hermann and Lecomte, 2019). FOSC strains exhibit strong host specificity toward a limited number of plant species, although some strains from different evolutionary lineages can infect the same host plants (Ma et al., 2013). During the infection process, soil-inhabiting FOSC fungi adhere to the plant root surface, undergo hyphal growth, penetrate the root epidermis, and eventually colonize the xylem vessels (Di Pietro et al., 2003; Michielse and Rep, 2009) This colonization disrupts various physiological, biochemical, and metabolic events, leading to wilting and eventual death of the invaded plants.

In the past two decades, extensive research has focused on understanding the molecular mechanisms underlying pathogenicity of *F. oxysporum* during its interactions with tomato or *Arabidopsis*. Throughout the infection process, *F. oxysporum* induces alterations in extracellular and intracellular pH (Masachis et al., 2016; Fernandes et al., 2023), stimulates the production of reactive oxygen species (ROS) by the NADPH oxidase B complex to facilitate chemotropic growth (Nordzieke et al., 2019), and neutralizes plant-derived ROS (Zhang et al., 2023). Simultaneously, *F. oxysporum* secretes small effector proteins into the xylem, collectively known as Secreted in Xylem (SIX), to disrupt and circumvent plant immunity, thereby enhancing the fungal pathogenicity (de Sain and Rep, 2015; Jangir et al., 2021). Researchers have characterized 14 *SIX* genes in *F. oxysporum* f. sp. *lycopersici* (Houterman et al., 2007; de Sain and Rep, 2015). Among these, *FolSIX1*, *FolSIX3*, *FolSIX5*, *FolSIX6*, and *FolSIX8* are crucial for full pathogenicity of *F. oxysporum* f. sp. *lycopersici* in susceptible tomato plants (Jangir et al., 2021). Furthermore, extensive studies have been conducted on various pathogenicity-related components, including transcription factors, governing pathogenicity functions in *F. oxysporum* (Husaini et al., 2018; Zuriegat et al., 2021). For instance, FoCon7-1, FoSge1, FoFtf1, Folctf1, Folctf2, and FolCzf1 play critical roles in *F. oxysporum* pathogenicity, likely by regulating the expression of effector genes (Michielse et al., 2009; Bravo-Ruiz et al., 2013; Ruiz-Roldán et al., 2015; Yun et al., 2019). Despite these significant advances, a comprehensive understanding of the molecular mechanisms and regulatory network governing *F. oxysporum* pathogenicity on distinct host plants remains to be fully established.

Post-translational modifications (PTMs), including phosphorylation, ubiquitination, glycosylation, acetylation, and lipidation, are pivotal for regulating the development, stress responses, and pathogenicity of plant pathogenic fungi. These PTMs profoundly influence the biochemical functions of proteins, affecting their activity, stability, and subcellular localization (Liu et al., 2021). For instance, *N*-glycosylation, catalyzed by *N*-glycosyltransferase Gnt2, is indispensable for the morphogenesis and virulence of *F. oxysporum* (Lopez-Fernandez et al., 2015). Additionally, palmitoyl transferase FonPAT2-catalyzed palmitoylation of the FonAP-2 complex influences the stability and interactions among core subunits of the complex, playing a vital role in growth, development, stress responses, and pathogenicity of *F. oxysporum* (Xiong et al., 2023). Lysine acetyltransferase FolArd1-mediated acetylation regulates the stability of the effector FolSvp1, thereby implicating its role in the pathogenicity of *F. oxysporum* (Li et al., 2022). Further exploration and characterization of various PTMs on pathogenicity-related proteins will undoubtedly yield new insights, shedding light on the molecular mechanisms underpinning *F. oxysporum* pathogenicity.

Poly(ADP-ribosyl)ation (PARylation), a PTM process, was initially discovered in the 1960s (Chambon et al., 1966; Hasegawa et al., 1967) and has since been found ubiquitous in various organisms (Alemasova and Lavrik, 2019). PARylation primarily relies on poly(ADP-ribose) polymerases (PARPs), which transfer ADP-ribose moieties from nicotinamide adenine dinucleotide (NAD^+^) to target proteins or themselves, resulting in the formation of linear or branched poly(ADP-ribose) polymers on glutamate (Glu), aspartate (Asp) or lysine (Lys) residues (D’Amours et al., 1999; Gibson and Kraus, 2012; Bai, 2015). Notably, lysine at 988 position (E988) of PARP1 is an important site that is associated with the occurrence of self-PARylation and mutation of this lysine led to a failure in creating PAR chain elongation, only adding a single mono-ADP-ribosyl (MAR) to the site (Chen et al., 2019). Conversely, these covalently attached polymers can be enzymatically degraded by poly(ADP-ribose) glycohydrolase (PARG) through endo- and exo-glycosidase reactions (Meyer-Ficca et al., 2004). PARylation has been confirmed to play essential roles in numerous cellular processes, including DNA repair, transcription regulation, chromatin modification, and ribosome biogenesis (Gibson and Kraus, 2012; Bock et al., 2015; Dasovich and Leung, 2023). Moreover, protein PARylation has implications in plant immunity against different pathogens (Feng et al., 2015; Song et al., 2015). For example, disrupting *AtPARPs* or *AtPARGs* modifies *Arabidopsis* responses to biotic and abiotic stresses (Adams-Phillips et al., 2010; Feng et al., 2015; Song et al., 2015, Yao et al., 2021). However, in filamentous fungi, PrpA, a putative PARP homolog in *Aspergillus nidulans*, functions early in the DNA damage response (Semighini et al., 2006). It was previously shown that knocking out *FolPARG1* had no discernible effect on the pathogenicity of *F. oxysporum* f. sp. *lycopersici* (Araiza-Cervantes et al., 2018). To date, the biological functions of PARPs and protein PARylation in plant pathogenic fungi remain largely unexplored.

Kin4, a protein kinase, functions as a mother cell-specific SPOC (spindle position checkpoint) component. It plays crucial role in regulating the MEN (mitotic exit network) activity and contributes to delaying cell cycle progression with spindle misalignment (D’Aquino et al., 2005; Pereira and Schiebel, 2005). In the yeast *Saccharomyces cerevisiae*, the deletion of *ScKin4* reduces the survival of *Δkar9* cells and exhibits a modest impact on mitotic progression under normal growth conditions (Tong et al., 2004; D’Aquino et al., 2005). In the filamentous fungus *Aspergillus nidulans*, KfsA, a homolog of ScKin4, proves essential for proper asexual spore formation (Takeshita et al., 2007). As Kin4 is a protein kinase, it is noteworthy that ScKin4 possesses the ability to phosphorylate with a catalytic site T209 located in the active loop of ScKin4 (D’Aquino et al., 2005; Caydasi et al., 2014). However, the biological functions of Kin4 and its biochemical relationship with PARP1 in plant pathogenic fungi remain poorly understood.

*Fusarium oxysporum* f. sp. *niveum* (*Fon*) causes devastating vascular Fusarium wilt disease, posing a significant threat to the global watermelon industry (Martyn, 2014; Everts and Himmelstein, 2015). In this study, our objective was to explore the functions of PARylation-related enzymes, FonPARP1 and FonPARG1, in *Fon* pathogenicity and elucidate their interplay with FonKin4. Our results demonstrate that both FonPARP1 and FonKin4 play critical roles in *Fon* pathogenicity, with FonKin4-mediated phosphorylation of FonPARP1 being a key regulatory mechanism. These findings underscore the significance of protein PARylation, facilitated by the FonKin4-FonPARP1 cascade, within the regulatory network governing pathogenicity in *Fon* and other plant pathogenic fungi.

## 2 Materials and methods

### 2.1 Fungal strains and growth conditions

The wild-type (WT) strain, *Fon* race 1 strain ZJ1, was used for generating deletion mutants (Gao et al., 2022). Experiments related to fundamental biological processes and stress responses were conducted as previously reported (Dai et al., 2016). For assessing growth, *Fon* strains were cultivated on potato dextrose agar medium (PDA) or minimal medium (MM) at 26°C for 7 d (Gao et al., 2022). Conidiation and germination assays were performed using mung bean liquid (MBL) and yeast extract peptone dextrose (YEPD) at 26°C for 2 d and 12 h, respectively (Gao et al., 2022). To observe conidial morphology and septation, macroconidia were stained with 10 μg/mL calcofluor white (CFW) and observed under a Zeiss LSM780 confocal microscope (Gottingen, Niedersachsen, Germany) (Gao et al., 2022). For stress response assays, *Fon* strains were cultivated on PDA supplemented with 0.7 M sodium chloride (NaCl; Sinopharm Chemical, Shanghai, China), 0.7 M calcium chloride (CaCl_2_; Sinopharm Chemical), 3 mM paraquat (Syngenta Crop Protection, Basel, Switzerland), 5 mM hydrogen peroxide (H_2_O_2_; Sigma-Aldrich, St. Louis, MO, United States), 0.2 g/L Congo red (CR; Sigma-Aldrich), 0.2 g/L Calcofluor white (CFW; Sigma-Aldrich) or 0.3 g/L sodium dodecyl sulfate (SDS; Sigma-Aldrich) for 7 d. The mycelial growth inhibition rate (MGIR) was calculated as described previously (Tang et al., 2018).

### 2.2 Generation of targeted deletion mutants and complementation strains

Deletion and complementation vectors were constructed as previously described (Gao et al., 2022). To generate targeted deletion mutants, the upstream and downstream flanking sequences of the target genes were amplified and then fused with the *HPH* (Hygromycin B phosphotransferase) fragment through a double-joint polymerase chain reaction (PCR). The PCR products were subsequently transformed into WT protoplasts. Transformants were selected on PDA containing 100 μg/mL hygromycin B (Roche, Basel, Switzerland) and further verified through PCR and Southern blotting. To generate complementation vectors, genomic fragments containing ~1.5 Kb native promoter and open reading frame (without a stop codon) of *FonPARP1*, *FonKin4*, and their mutated variants were co-transformed with *Xho*I-digested pYF11-neo plasmid into the yeast strain XK-125 using the Alkali-Cation Yeast Transformation Kit (MP Biomedicals, Solon, OH, United States). The resulting recombinant vectors were introduced into protoplasts of the corresponding deletion mutants. Transformants were selected on PDA supplemented with 50 μg/mL neomycin (Sangon Biotech, Shanghai, China) and confirmed by PCR amplification or Western blotting with anti-GFP antibody (Cat. #ab290, Abcam, Cambridge, United Kingdom) or anti-GAPDH antibody (Cat. #EM1101, HuaBio, Hangzhou, China). Site-specific point mutated variants FonPARP1^E729K^ and FonKin4^T462A^ were created using Mut Express MultiS Fast Mutagenesis Kit (Vazyme Biotech, Nanjing, China).

### 2.3 Disease assays and fungal biomass estimation

Disease assays were performed using a previously established protocol (Dai et al., 2016). Watermelon (*Citrullus lanatus* L. cv. Zaojia) plants were grown in a potting mix (vermiculite: plant ash: perlite = 6:2:1) in a growth room with a 16-h light/8-h dark photoperiod. For inoculation, three-week-old plant roots were dipped in spore suspensions (5 × 10^6^ spores/mL) of different *Fon* strains for 15 min, subsequently replanted in soil, and covered with plastic wrap for 3 d. Disease symptoms and progress were assessed using a 4-scale rating standard (0 = no symptom, 1 = chlorosis, 2 = wilting, 3 = death). For tissue examination, 1 cm segments of roots and stems were collected from the inoculated plants at 15 d post-inoculation, sterilized by immersion in 70% ethanol for 30 s, and then placed on PDA for incubation at 26°C for 3 d (Gao et al., 2023). Colony morphology, mycelial color and conidia characteristics were carefully examined to distinguish *Fon* and other fungal contamination. To estimate *in planta* fungal biomass, three-week-old plants were cultivated in spore suspensions of different *Fon* strains with shaking (85 rpm). Samples were collected at different time points post-inoculation and qRT-PCR was conducted to determine the levels of *FonOpm12* and watermelon *ClRps10* (used as an internal control). Relative fungal biomass was expressed as the *FonOpm12*/*ClRps10* ratio (Gao et al., 2022).

### 2.4 RNA extraction and reverse transcriptase (RT)-qPCR

Total RNA was extracted from mycelia cultured in PDB for 2 d using RNA Isolater reagent (Vazyme Biotech). First-strand cDNA was synthesized using HiScript II qRT SuperMix kit (Vazyme Biotech) following the manufacturer’s recommendations. Reactions for qPCR were prepared with 2× AceQ qPCR SYBR Green Master Mix (Vazyme Biotech) and run on a LightCycler 96 instrument (Roche). *FonActin* served as an internal control to normalize the qPCR data and relative expression levels of genes were calculated using the 2^–△△CT^ method. The primers used are listed in Supplementary Table S1.

### 2.5 Microscopic examinations

To observe subcellular localization, fresh mycelia and macroconidia of WT::FonKin4-GFP strain were harvested from two-day-old culture grown on potato dextrose broth (PDB) at 26°C and then examined under a Zeiss LSM780 confocal microscope using the appropriate conditions for capturing the GFP signal.

### 2.6 Yeast two-hybrid (Y2H) assays

Y2H assays were conducted following the manufacturer’s instructions for the Matchmaker Gold Y2H System (Clontech, Mountain View, CA, United States) with the yeast strain Y2H Gold. The coding sequences of full-length FonKin4 and the N-terminal region of FonPARP1 were amplified with gene-specific primers (Supplementary Table S1) and cloned into double-cleaved pGBKT7 and pGADT7 using the principle of homologous recombination, respectively. Paired vectors were co-transformed through LiAc/SS carrier DNA/PEG method (Gietz and Schiestl, 2007). After incubation on a basic medium (SD) lacking Leu and Trp at 30°C for 5 d, the transformants were screened on SD/−Leu/−Trp/-His/−Ade medium supplemented with 40 μg/mL X-α-gal (Clontech, Mountain View, CA, United States).

### 2.7 Co-immunoprecipitation (Co-IP) assays

The coding sequences of the targeted genes were incorporated into pYF11 vector, featuring a C-terminal GFP tag, and/or pHZ126 vector, harboring a 3× FLAG tag (Yin et al., 2020). Subsequently, these constructs were transformed into WT protoplasts, and the transformants were grown on PDA containing hygromycin B and/or neomycin, followed by confirmation using PCR amplification and Western blotting. For Co-IP assays, mycelia cultured in PDB for 2 d were harvested. Total proteins were extracted using a lysis buffer (1 M Tris–HCl, pH7.4, 0.5 M EDTA, 1 M NaCl, 0.1% Triton X-100, 1 mM DTT, and 1× protease inhibitor cocktail) and precipitated with GFP-Trap beads (ChromoTek, Planegg-Martinsried, Germany) following the manufacturer’s instructions. The eluted proteins were subsequently detected by immunoblotting via using either anti-GFP antibody or anti-FLAG antibody (Cat. # A8592, Sigma-Aldrich), respectively.

### 2.8 Purification of recombinant proteins

The coding sequences of *FonPARP1*, *FonKin4*, and *FonKin4-ST* were cloned into the pGEX4T-3 vector, featuring a GST tag, or pET32a vector, possessing an HIS tag. Prokaryotic expression and purification of recombinant proteins were performed as previously described (Liu et al., 2019). Briefly, *E. coli* Rosetta cells carrying the recombinant vectors were induced by 1 mM Isopropyl ß-D-1-thiogalactopyranoside (IPTG; Sigma-Aldrich) at 18°C for 20 h. Subsequently, the recombinant proteins were purified using a GST fusion protein purification kit (Genscript, Piscataway, NJ, United States) and Profinity IMAC Ni-charged resin (Bio-Rad, Hercules, CA, United States), respectively.

### 2.9 *In vitro* pull-down assays

GST-tagged recombinant proteins were immobilized on glutathione-Sepharose beads (Yeasen, Shanghai, China) and then incubated with HIS-tagged recombinant proteins in GST buffer at 4°C for 3 h. The eluted proteins were then separated on a 12.5% SDS-PAGE and immunoblotted with anti-GST antibody (Cat. #A00865, GenScript) or anti-HIS antibody (Cat. #A00186, GenScript), respectively.

### 2.10 Western blotting assays

Western blotting assay was carried out as previously described (Gao et al., 2022). Samples were separated on SDS-PAGE gels of varying percentages (8%, 12.5% or 15%) and subsequently transferred onto an Immobilon-P transfer membrane (Millipore, Billerica, MA, United States). The membranes were then immunoblotted with appropriate antibodies. Blot detection was accomplished using the ECL chromogenic reagent (Thermo Fisher Scientific, Waltham, MA, United States), and the bands were scanned or imaged using the Tanon automatic gel imaging system (Tianneng Corporation, Shanghai, China).

### 2.11 Immunoprecipitation-liquid chromatography–tandem mass spectrometry (IP-LC–MS/MS)

The FonPARP1-GFP strain was cultured in PDB for 2 d, and total proteins were extracted from fresh mycelial samples using above-mentioned lysis buffer. Subsequently, proteins were precipitated with GFP-Trap beads following the manufacturer’s instructions, eluted using the elution buffer (100 mM Tris–HCl, pH7.6, 4% SDS, and 1 mM DTT), and subjected to LC–MS/MS analysis. MS/MS spectra was searched using MASCOT engine (Matrix Science, London, United Kingdom; version 2.2) against *Fusarium oxysporum* f. sp. *lycopersici* 4,287 protein sequences database (NCBI). For protein identification, the following options were used. The peptide mass tolerance was set to 20 ppm, and the MS/MS fragment tolerance was set to 0.1 Da. The enzyme was trypsin. The missed cleavage was set to 2. The ion score of peptides was set to ≥20.

### 2.12 *In vitro* phosphorylation assays

*In vitro* phosphorylation assays were conducted as previously reported (Xia et al., 2021). Briefly, 2 μg HIS-FonKin4 with or without 4 μg HIS-FonPARP1 were incubated in a 50 μL kinase reaction buffer (20 mM HEPES-KOH, 10 mM MgCl_2_, 40 mM ATP, 25× protein inhibitor cocktail, pH7.5) at 30°C for 1 h. After incubation, the reaction was halted by adding 4× SDS loading buffer. Subsequently, the proteins were separated on 8% SDS-PAGE and detected using anti-phosphor Ser/Thr antibody (Cat. #PP2551, ECM Biosciences, Aurora, CO, United States) or anti-HIS antibody (Cat. #A00186, GenScript).

### 2.13 *In vitro* PARylation assays

*In vitro* PARylation assays were performed as described previously (Kong et al., 2021; Yao et al., 2021). Briefly, 500 ng HIS-FonPARP1 were incubated in a 50 μL PARylation buffer (50 mM Tris–HCl, 50 mM NaCl, 10 mM MgCl_2_, pH8.0) with 0.2 mM NAD^+^, 1× activated DNA (BPS Bioscience, San Diego, CA, USA), and 3 μg GST-FonKin4 at 26°C for 3 h. Subsequently, the samples were separated on 8% and 12.5% SDS-PAGE. PARylated proteins were detected via immunoblotting with anti-poly-ADPR antibody (Cat. #MABE1031, Sigma-Aldrich), anti-GST antibody (Cat. #A00865, GenScript) or anti-HIS antibody (Cat. #A00186, GenScript).

For *in vitro* phosphorylation-mediated self-PARylation assays, 4 μg HIS-FonPARP1 underwent prior *in vitro* phosphorylation by HIS-FonKin4-ST, as described above. Then, 50 μL PARylation buffer was then added, followed by incubation at 26°C for 30 min. The reaction was stopped by adding 4× SDS loading buffer, and the proteins were then separated on 8% and 12.5% SDS-PAGE. Phosphorylated proteins were detected by anti-phosphor Ser/Thr antibody. PARylated proteins detected through immunoblotting with streptavidin-HRP (Cat. #21126, Thermo Fisher Scientific, Waltham, MA, USA) for Biotin NAD^+^ or anti-HIS antibody (Cat. #A00186, GenScript).

### 2.14 Phylogenetic analysis

Protein sequences were searched from National Center for Biotechnology Information (NCBI) using HMMER (Hidden Markov Model search, Hmmsearch) web server as a query. Sequences were initially aligned using CLUSTALX. Subsequently, phylogenetic analysis was conducted with MEGA7 software using the neighbor-joining method based on boostrap = 1,000. SMART protein database (http://smart.embl-heidelberg.de/) was utilized to analyze protein conserved domains.

### 2.15 Statistical analysis

All experiments were conducted independently three times. The data were subjected to statistical analysis using either the Student’s *t*-test or one-way analysis of variance (ANOVA). Significant differences were defined by probability values of *p* < 0.05 or *p* < 0.01.

## 3 Results

### 3.1 Characterization of FonPARP1 and FonPARG1 in *Fon*

To investigate the biological functions of PARylation in *Fon*, we initially searched for genes encoding PARylation-related PARP and PARG enzymes. HMMER was utilized to query the *F. oxysporum* f. sp. *lycoperici* reference genome database using *Arabidopsis* AtPARP and AtPARG protein sequences as references (Prakash et al., 2017). This analysis led to the characterization of two genes (FOXG_07574 and FOXG_05947) that potentially encode PARP and PARG enzymes in *F. oxysporum* (Supplementary Figure S1A), respectively. We confirmed the sequences for the predicted open reading frames (ORF) of these two genes through cloning and sequencing from *Fon*, subsequently naming them *FonPARP1* and *FonPARG1*. FonPARP1 exhibited close relatedness to its orthologues in *F. oxysporum* f. sp. *melonis*, *F. verticillioides*, and *F. graminearum* but was distantly related to those in *Magnaporthe oryzae*, *Aspergillus nidulans*, and *Neurospora crassa* (Supplementary Figure S1B). FonPARP1, comprising 748 amino acids, featured BRCT and WGR domains at its N-terminus as well as PARP regulatory and PARP catalytic domains at its C-terminus (Supplementary Figure S1C). This structural arrangement exhibited high conservation across various fungi (Supplementary Figure S2). FonPARG1 clustered with FomPARG1 from *F. oxysporum* f. sp. *melonis* (Supplementary Figure S1D) and encompassed 476 amino acids, characterized by a single conserved PARG catalytic domain (Supplementary Figures S1A,E, S3). Overall, the conservation of FonPARP1 and FonPARG1 across multiple fungi, along with the identification of their critical structural domains, suggests their likely conservation of function.

### 3.2 Generation of targeted deletion mutants and complementation strains for *FonPARP1* and *FonPARG1*

To elucidate the biological functions of *FonPARP1* and *FonPARG1* in *Fon*, we generated targeted deletion mutants *ΔFonPARP1* and *ΔFonPARG1* employing the homologous recombination strategy (Supplementary Figures S4A,B). These deletion mutants were verified by Southern blotting using an *HPH* probe, confirming the presence of a single insertional *HPH* fragment in the mutants but not in the WT strain (Supplementary Figures S4C,D). RT-qPCR results revealed that the transcripts of *FonPARP1* and *FonPARG1* were barely detectable in the respective deletion mutants (Supplementary Figures S4E,F). To construct a complementation strain *ΔFonPARP1*-C, we expressed a native promoter-driven *FonPARP1-GFP* cascade in *ΔFonPARP1* background (Supplementary Figure S4G). Considering the importance of glutamic acid (E) at 988 position for the enzymatic activity of human PARP1 (Altmeyer et al., 2009; Chen et al., 2019), we created a mutated variant FonPARP1^E729K^, in which conserved E residue was replaced with lysine (K). This variant was fused with a GFP tag and introduced into *ΔFonPARP1* to generate a mutated complementation strain *ΔFonPARP1*-C^E729K^ (Supplementary Figure S4G). Since various lines of the mutants and complementation strains exhibited comparable phenotypes, we selected one representative line of *ΔFonPARP1*, *ΔFonPARG1*, *ΔFonPARP1*-C, and *ΔFonPARP1*-C^E729K^ for further investigations.

### 3.3 FonPARP1 is required for *Fon* pathogenicity

**Figure 1:**
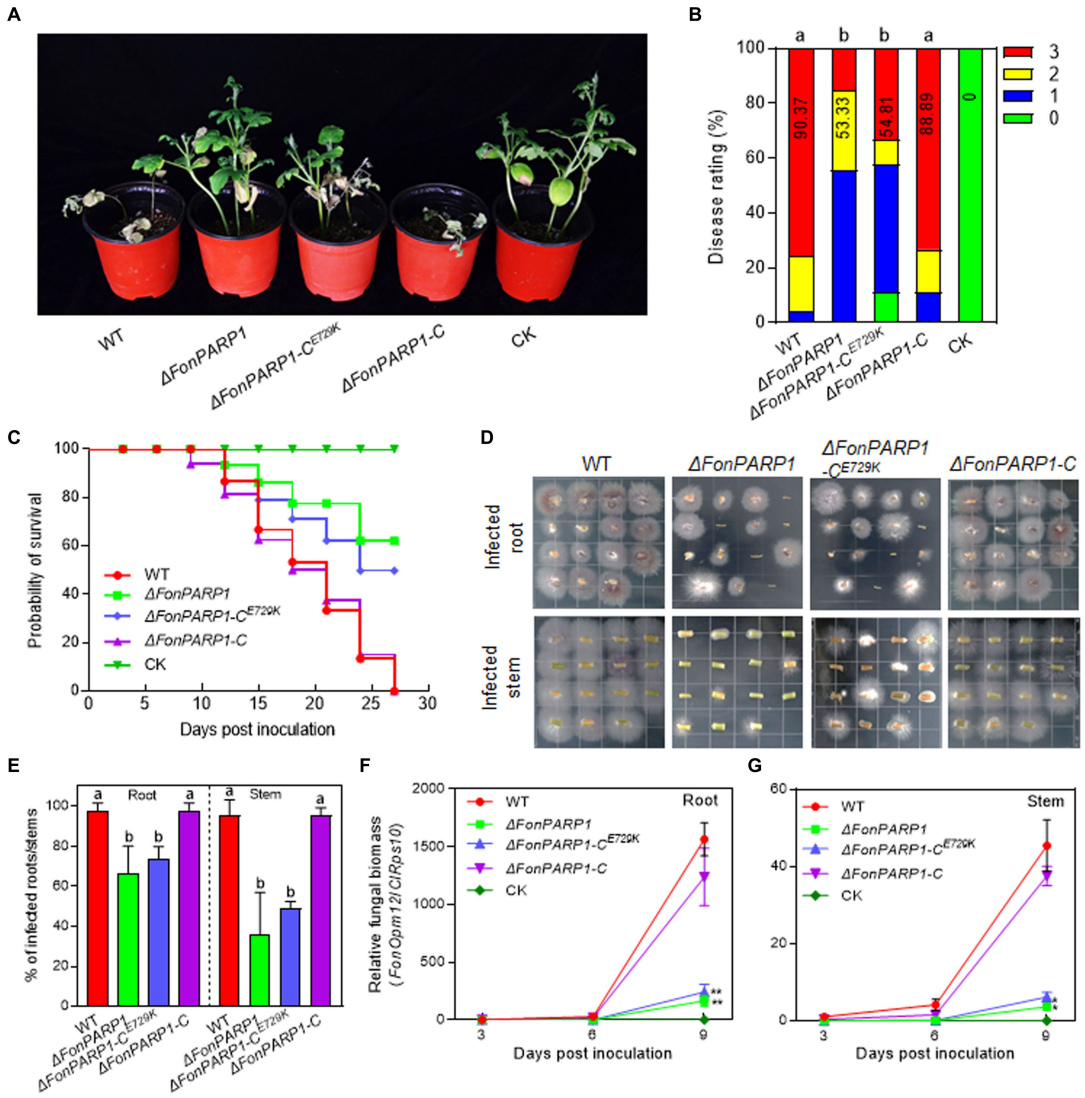
FonPARP1 is required for *Fon* pathogenicity on watermelon. **(A,B)** Disease phenotype **(A)** and disease ratings **(B)** of watermelon plants inoculated with WT, *ΔFonPARP1*, *ΔFonPARP1*-C^E729K^ or *ΔFonPARP1*-C strains at 21 dpi. **(C)** Survival curves of watermelon plants inoculated with WT, *ΔFonPARP1*, *ΔFonPARP1*-C^E729K^ or *ΔFonPARP1*-C strains during a 27-d experimental period. **(D,E)** Tissue examination **(D)** and percentages **(E)** of fungal colonies grown from roots and stems of WT-, *ΔFonPARP1*-, *ΔFonPARP1*-C^E729K^- or *ΔFonPARP1*-C-inoculated watermelon plants at 15 dpi. **(F,G)** Relative *in planta* fungal biomass of WT, *ΔFonPARP1*, *ΔFonPARP1*-C^E729K^, and *ΔFonPARP1*-C in roots and stems of the inoculated plants. Relative fungal biomass was quantified by qRT-PCR and is presented as the ratio of *FonOpm12*/*ClRps10*. Experiments were independently performed three times with similar results. Data presented in **(B–G)** represent the means ± SD from three independent experiments. Different letters in **(B,E)** or asterisks in **(F,G)** indicate significant differences at *p* < 0.05 level by one-way ANOVA or Student’s *t*-test, respectively.

We then investigated the involvement of *FonPARP1* and *FonPARG1* in *Fon* pathogenicity by assessing the disease-causing ability of the deletion mutants *ΔFonPARP1*, *ΔFonPARG1* as well as their complementation strains. In repeated experiments, *ΔFonPARP1*-inoculated plants exhibited less severe disease symptoms, with 55% displaying cotyledon yellowing and slight wilting (Figures 1A,B). The disease ratings of these plants decreased by 41% compared to WT-inoculated plants (Figures 1A,B). Conversely, *ΔFonPARG1*-inoculated plants showed no significant difference in disease development compared to WT-inoculated plants (Supplementary Figures S5A,B). Notably, *ΔFonPARP1*-C^E729K^-inoculated plants presented similar disease symptoms to the *ΔFonPARP1*-inoculated plants, and the disease index was reduced by 39% compared to WT-inoculated plants (Figures 1A,B). These data suggest that *FonPARP1* is essential for *Fon* pathogenicity, whereby *FonPARG1* is not required, and emphasize the critical role of conserved E729 amino acid in the function of FonPARP1 in *Fon* pathogenicity.

To further confirm the function of *FonPARP1* and *FonPARG1* in *Fon* pathogenicity, we monitored disease progression in watermelon plants following inoculation with the deletion mutants and their corresponding complementation strains. We observed a delay of ~3 d in the onset of disease-caused death in plants inoculated with *ΔFonPARP1* and *ΔFonPARP1-*C^E729K^ compared with those inoculated with *ΔFonPARP1*-C, which began to die at 9 d post-inoculation (dpi) (Figure 1C). During a 27-d experiment period, the death rates progressed at a slower pace in plants inoculated with *ΔFonPARP1* and *ΔFonPARP1-*C^E729K^ than those inoculated with WT and *ΔFonPARP1*-C (Figure 1C). At 27 dpi, all WT- and *ΔFonPARP1*-C-inoculated plants had died, while only 38 and 50% of the *ΔFonPARP1*- and *ΔFonPARP1-*C^E729K^-inoculated plants succumbed to the disease, respectively (Figure 1C). In contrast, the disease progression and death rate of the *ΔFonPARG1*-inoculated plants were similar to those of the WT-inoculated plants (Supplementary Figure S5C), confirming that FonPARG1 is not required for *Fon* pathogenicity.

We conducted further examinations to evaluate the *in planta* fungal biomass of *ΔFonPARP1* and its complementation strains, *ΔFonPARP1-*C and *ΔFonPARP1-*C^E729K^, in inoculated plants. In tissue examination assays, root and stem segments from *ΔFonPARP1* and *ΔFonPARP1-*C^E729K^-infected plants supported fewer *Fon* colonies compared to segments from WT- and *ΔFonPARP1-*C-infected plants (Figure 1D), showing reductions of 25 ~ 31% in roots and 47 ~ 60% in stems (Figure 1E). Further, the relative *in planta* fungal biomass of *ΔFonPARP1* and *ΔFonPARP1-*C^E729K^ was obviously lower than that of WT and *ΔFonPARP1-*C in both roots and stems of the infected plants, displaying reductions of 85 ~ 90% in roots and 87 ~ 91% in stems compared to the WT strain at 9 dpi (Figures 1F,G). However, it is worth noting that *ΔFonPARP1* still exhibited the ability to penetrate the cellophane membrane, similar to WT and *ΔFonPARP1-*C (Supplementary Figure S6). Taken together, these results indicate that *FonPARP1* plays a vital role in *Fon* pathogenicity, likely by affecting the invasive growth within watermelon plants rather than influencing the penetration ability.

### 3.4 FonKin4 interacts with FonPARP1

**Figure 2:**
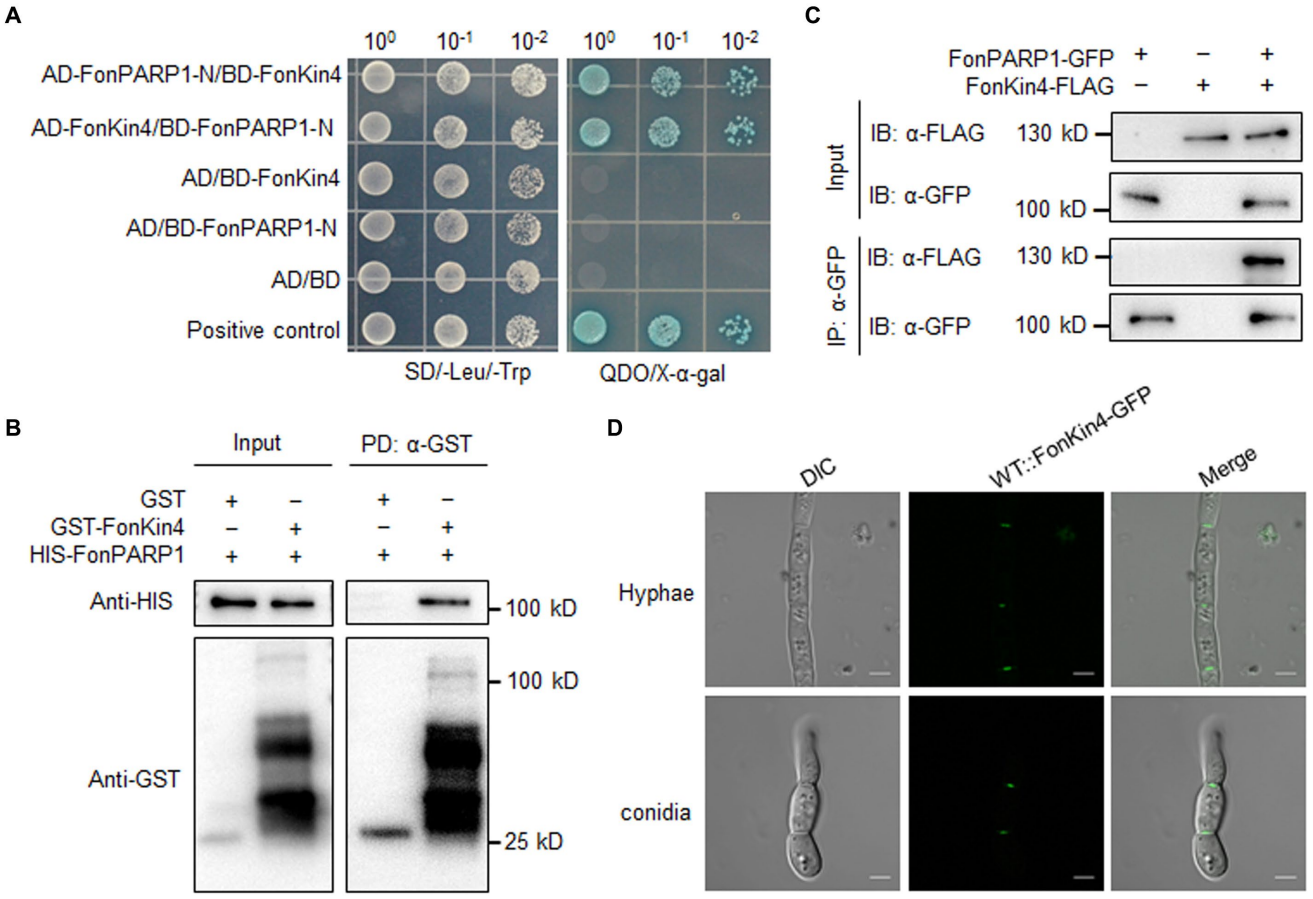
FonKin4 interacts with FonPARP1. **(A)** Interaction between FonKin4 and FonPARP1 in Y2H assays. Equal amounts of yeast cells expressing different combinations were grown on SD/−Leu/−Trp and QDO/X-α-gal plates. **(B)** Interaction between FonKin4 and FonPARP1 in pull-down assays. HIS-tagged FonPARP1 was incubated with immobilized GST-tagged FonKin4. The samples were detected by anti-HIS or anti-GST antibody. **(C)** Interaction between FonKin4 and FonPARP1 in Co-IP assays. FonPARP1-GFP and FonKin4-FLAG were co-expressed in WT strain. Total proteins were extracted, immunoprecipitated with GFP-Trap beads, and detected by anti-GFP or anti-FLAG antibody. **(D)** Subcellular localization of FonKin4-GFP in mycelia and macroconidia of *Fon*. Scale bar = 5 μm.

To explore the biochemical mechanism of FonPARP1 in regulating *Fon* pathogenicity, we sought to identify putative FonPARP1-interacting factors by performing LC–MS/MS characterization of FonPARP1-GFP-immunoprecipitated proteins. In this analysis, we detected 48 peptides corresponding to 53 genes that may encode putative FonPARP1-interacting factors (Supplementary Table S2). Among these, we shortlisted FOXG_01025, encoding a putative protein kinase consisting of 1,111 amino acids (Supplementary Figure S7A). Given that FOXG_01025 is very similar to yeast Kin4 (D’Aquino et al., 2005), we named it FonKin4. FonKin4 was phylogenetically clustered with homologs from other plant pathogenic filamentous fungi (Supplementary Figure S7B) and possesses a typical S_TKc domain with a conserved active threonine residue (Supplementary Figures S7C,D). To validate the direct interaction between FonPARP1 and FonKin4, we conducted Y2H and pull-down assays. Since the expression of whole PARP1 interfered with cell growth in yeast (La Ferla et al., 2015; Yao et al., 2021), we thus used the N-terminal region of FonPARP1 (FonPARP1-N) for the Y2H assays. FonKin4 exhibited interaction with the N-terminal region of FonPARP1 (Figure 2A). In pull-down assays, GST-FonKin4, but not bare GST, successfully pulled-down HIS-FonPARP1 (Figure 2B). Additionally, Co-IP assays involving strains co-expressing PARP1-GFP and FonKin4-FLAG confirmed the *in vivo* association between FonKin4 and FonPARP1, as FonKin4-FLAG was co-immunoprecipitated with FonPARP1-GFP (Figure 2C). Furthermore, subcellular localization assays showed that FonKin4-GFP was predominantly localized around septa in both mycelia and macroconidia of *Fon* (Figure 2D).

### 3.5 FonKin4 is required for *Fon* pathogenicity

To explore whether FonKin4 plays a role in mediating *Fon* pathogenicity, we created a targeted deletion mutant *ΔFonKin4* (Supplementary Figure S8A). The obtained deletion mutant was validated through Southern blotting, detecting a single insertional *HPH* fragment, which was absent in WT (Supplementary Figure S8B). Additionally, RT-qPCR analysis revealed a significant reduction in *FonKin4* transcript level in the mutant (Supplementary Figure S8C). Concurrently, we generated a complementation strain *ΔFonKin4*-C by expressing a native promoter-driven *FonKin4-GFP* fusion in *ΔFonKin4* (Supplementary Figure S8D). Notable, the threonine (T) residue at 209 position in the activation T-loop is crucial for the enzymatic activity of ScKin4 (D’Aquino et al., 2005; Caydasi et al., 2014). We thus created a kinase-dead variant FonKin4^T462A^, in which T462, corresponding to T209 in ScKin4, was substituted with alanine (A), and introduced it into *ΔFonKin4*, resulting in a complementation strain *ΔFonKin4*-C^T462A^, harboring a FonKin^T462A^-GFP fusion (Supplementary Figure S8D).

**Figure 3:**
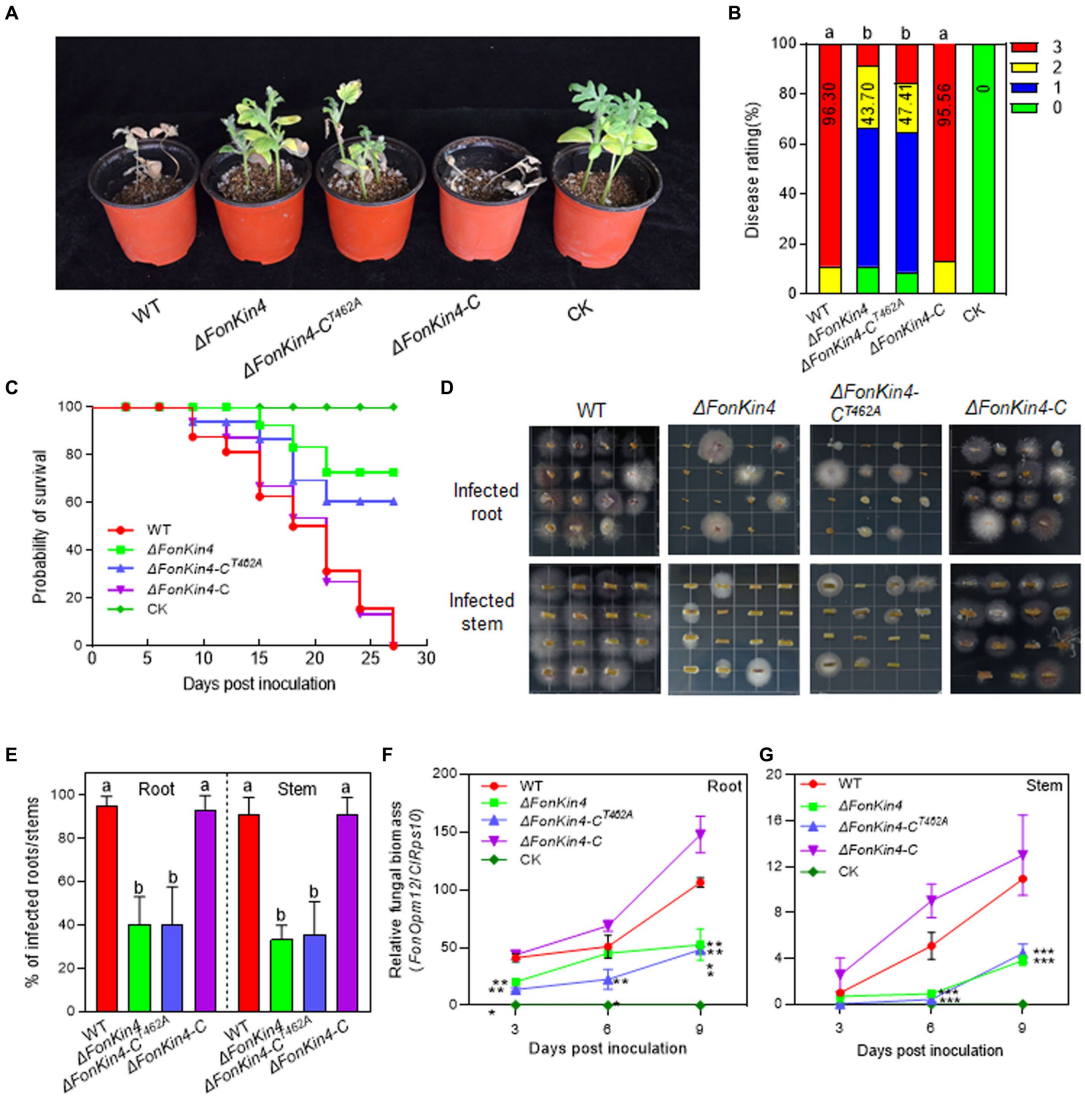
FonKin4 is required for *Fon* pathogenicity on watermelon. **(A,B)** Disease phenotype **(A)** and disease ratings **(B)** of watermelon plants inoculated with WT, *ΔFonKin4*, *ΔFonKin4*-C^T462A^ or *ΔFonKin4*-C strains at 21 dpi. **(C)** Survival curves of watermelon plants inoculated with WT, *ΔFonKin4*, *ΔFonKin4*-C^T462A^ or *ΔFonKin4*-C strains during a 27-d experimental period. **(D,E)** Tissue examination **(D)** and percentages **(E)** of fungal colonies grown from roots and stems of WT, *ΔFonKin4*, *ΔFonKin4*-C^T462A^ or *ΔFonKin4*-C-inoculated watermelon plants at 15 dpi. **(F,G)** Relative *in planta* fungal biomass of WT, *ΔFonKin4*, *ΔFonKin4*-C^T462A^ or *ΔFonKin4*-C in roots and stems of the inoculated plants. Relative fungal biomass was quantified by qPCR and is presented as the ratio of *FonOpm12*/*ClRps10*. Experiments were independently performed three times with similar results. Data presented in **(B–G)** represent the means ± SD from three independent experiments. Different letters in **(B,E)** or asterisks in **(F,G)** indicate significant differences at *p* < 0.05 level by one-way ANOVA or Student’s *t*-test, respectively.

To explore the involvement of *FonKin4* in *Fon* pathogenicity, we evaluated the disease-causing ability of the deletion mutant *ΔFonKin4* and its complementation strain. In repeated experiments, *ΔFonKin4*-inoculated plants displayed milder disease symptoms, with 11% remaining healthy and 56% exhibiting yellow cotyledons at 21 dpi (Figures 3A,B). Compared to the disease severity observed in WT-inoculated plants, the disease rating of *ΔFonKin4*-inoculated plants decreased by 55% (Figures 3A,B), while *ΔFonKin4*-C-inoculated plants exhibited comparable disease levels to WT-inoculated plants (Figures 3A,B). Notably, *ΔFonKin4*-C^T462A^-inoculated plants exhibited similar disease levels to those of *ΔFonKin4*-inoculated plants, leading to a 51% reduction in the disease index compared to WT-inoculated plants (Figures 3A,B). In disease progress monitoring experiments, we observed a delay of ~6 d in the onset of disease-induced mortality in *ΔFonKin4*-inoculated plants compared to WT- and *ΔFonKin4*-C-inoculated plants (Figure 3C). The death rates progressed more slowly in *ΔFonKin4*- and *ΔFonKin4*-C^T462A^-inoculated plants compared to WT- and *ΔFonKin4*-C-inoculated plants (Figure 3C). At 27 dpi, all WT- and *ΔFonKin4*-C-inoculated plants had succumbed to the disease, while ~72% and ~ 61% of *ΔFonKin4*- and *ΔFonKin4*-C^T462A^-inoculated plants remained alive, respectively (Figure 3C).

In tissue examination assays, root and stem segments from the *ΔFonKin4*- and *ΔFonKin4-*C^T462A^-infected plants displayed reduced *Fon* colonies compared to those from the WT- and *ΔFonKin4*-C-infected plants (Figure 3D), showing reductions of 54 ~ 55% in roots and 55 ~ 58% in stems (Figure 3E). Moreover, the relative *in planta* fungal biomass of *ΔFonKin4* and *ΔFonKin4*-C^T462^ was significantly lower compared to the WT and *ΔFonKin4-*C in both roots and stems of the infected plants. Specifically, the relative *in planta* fungal biomass decreased by 51% ~ 55% in roots and 59 ~ 65% in stems of the *ΔFonKin4*- and *ΔFonKin4*-C^T462^-inoculated plants compared to the WT-inoculated ones at 9 dpi (Figures 3F,G). Interestingly, the penetration ability of *ΔFonKin4* through the cellophane membrane was similar to that of the WT and *ΔFonKin4-*C (Supplementary Figure S6). Collectively, these results indicate that, similar to its interacting partner FonPARP1, FonKin4 plays a functional role in *Fon* pathogenicity by affecting the invasive growth within watermelon plants rather than influencing the penetration ability, and the conserved T462 residue is critical for FonKin4 function in *Fon* pathogenicity.

### 3.6 FonKin4 and FonPARP1 are involved in the basic biological processes of *Fon*

**Figure 4:**
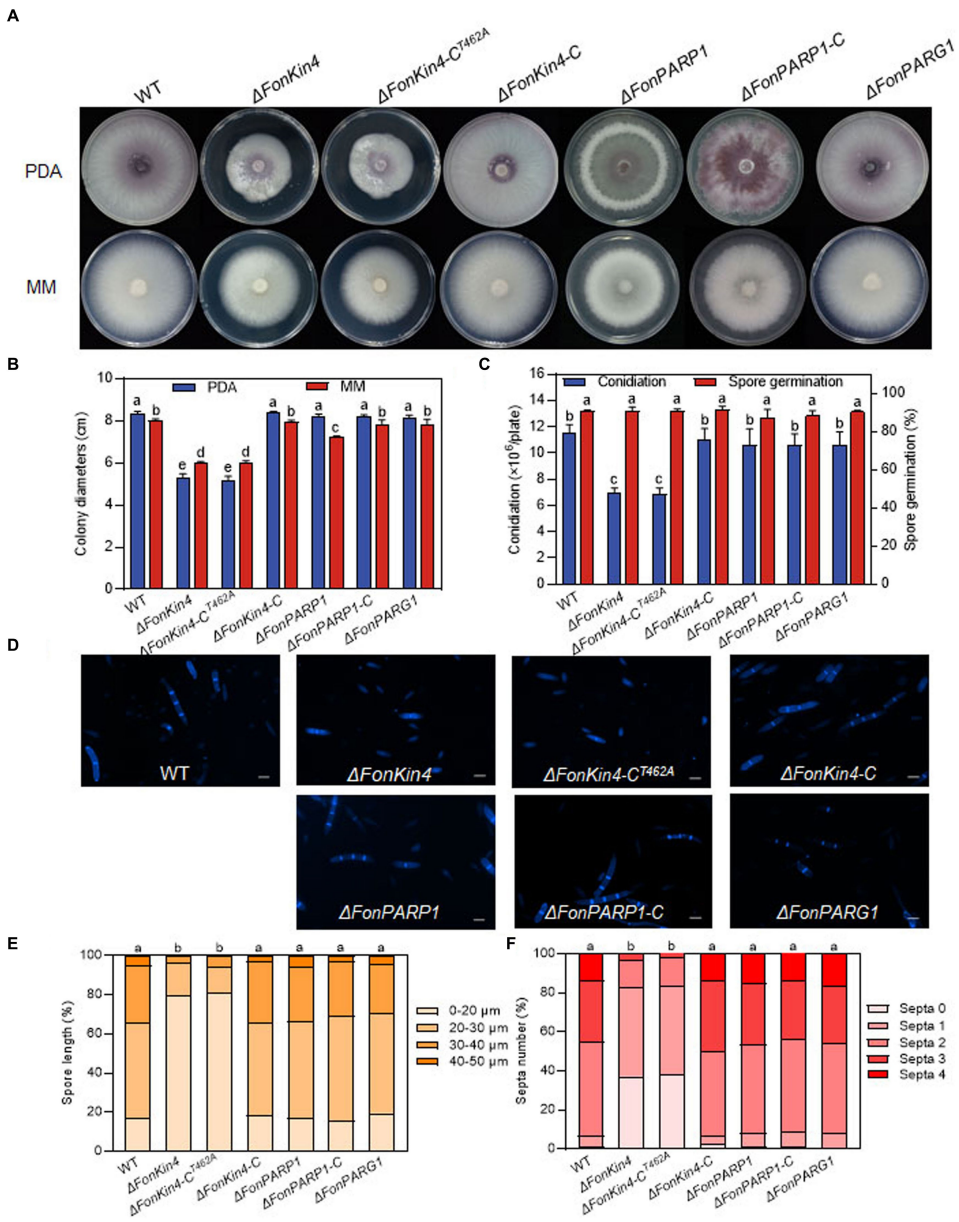
FonKin4 and FonPARP1 are involved in regulating the basic biological processes of *Fon*. **(A,B)** Mycelial growth **(A)** and colony diameters **(B)** of different strains grown on PDA and MM plates at 7 d. **(C)** Conidiation and spore germination of different strains. **(D–F)** Morphology **(D)**, length **(E)**, and septum numbers **(F)** of macroconidia produced by different strains. Scale bar = 5 μm. Experiments were independently performed three times with similar results. Data presented in **(B,C,E,F)**, represent the means ± SD from three independent experiments, and different letters indicate significant differences (*p* < 0.05, one-way ANOVA).

We also investigated whether FonPARP1, FonPARG1, and FonKin4 are involved in the basic biological processes of *Fon*. In repeated experiments, *ΔFonPARG1* showed similar phenotypes to WT regarding mycelial growth, conidiation, spore germination, and macroconidia morphology (Figures 4A–F), indicating that FonPARG1 is not involved in these basic biological processes of *Fon*. Moreover, *ΔFonPARP1* displayed slower growth on MM than WT and *ΔFonPARP1-*C (Figures 4A,B), while remained no changes in growth on PDA or other basic biological processes of *Fon* (Figures 4A–F). However, *ΔFonKin4* and *ΔFonKin4*-C^T462A^ exhibited slower growth on both PDA or MM (Figures 4A,B) and produced fewer macroconidia, with a reduction of ~40% compared to WT and *ΔFonKin4-*C (Figure 4C). The macroconidia produced by *ΔFonKin4* and *ΔFonKin4*-C^T462A^ germinated normally (Figure 4C) but displayed abnormal morphology with fewer septa and shorter lengths than those of WT and *ΔFonKin4-*C (Figures 4D–F). Particularly, ~80% of the *ΔFonKin4*- and *ΔFonKin4*-C^T462A^-produced macroconidia were shorter than 20 μm and had at most 1 septum, whereas >85% of macroconidia produced by WT and *ΔFonKin4-*C were longer than 20 μm and had more than 2 septa (Figures 4D–F). These results suggest that FonKin4 and its intact kinase activity play key roles in regulating vegetative growth, conidiation, and conidial morphology of *Fon*, while FonPARP1 affects mycelial growth under nutrient-scarce conditions.

### 3.7 FonPARP1, FonPARG1, and FonKin4 are involved in the abiotic stress responses of *Fon*

**Figure 5:**
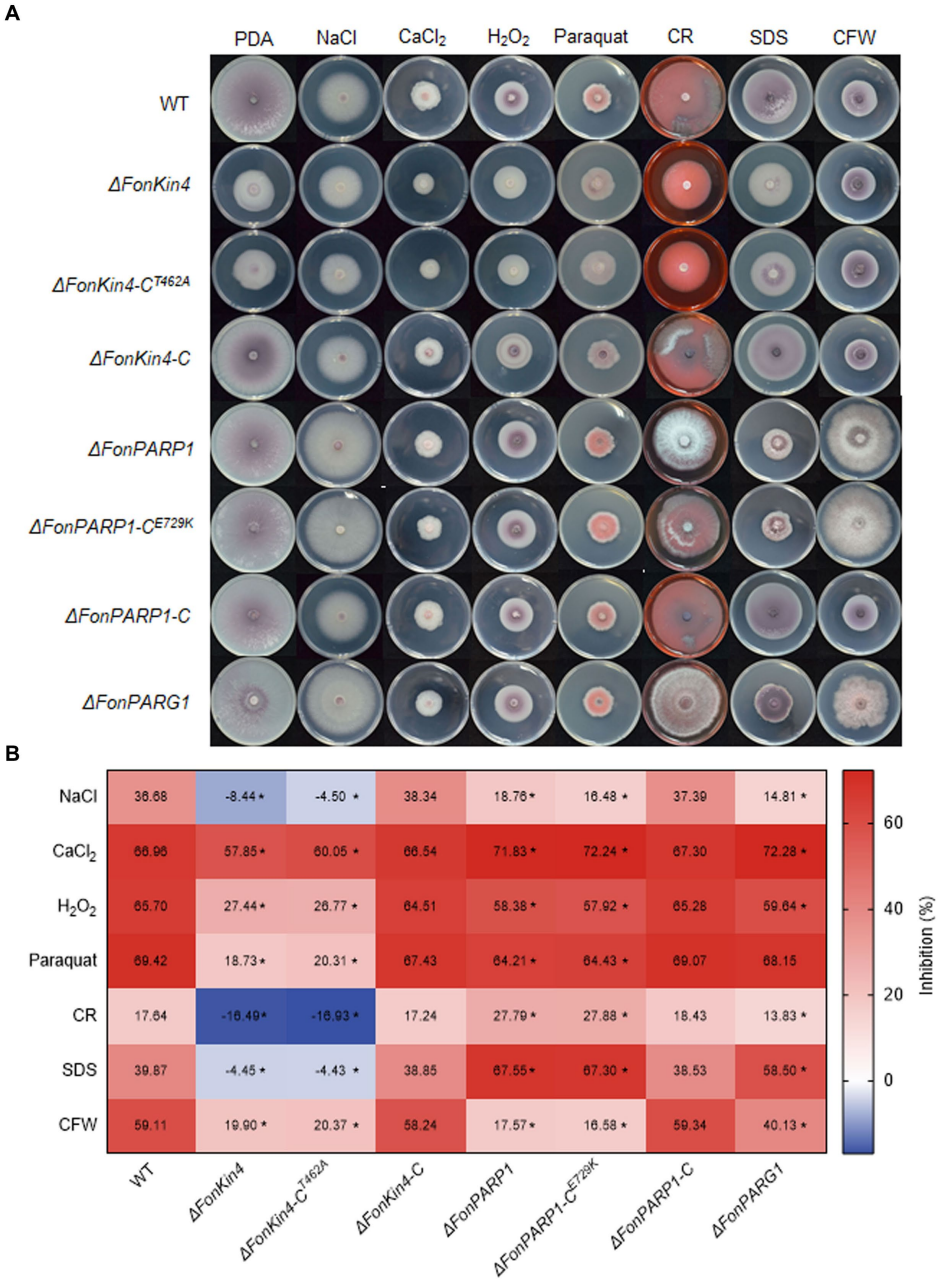
FonPARP1, FonPARG1, and FonKin4 are involved in the abiotic stress responses in *Fon*. **(A)** Mycelial growth and **(B)** inhibition rates of WT, *ΔFonKin4*, *ΔFonKin4*-C^T462A^, *ΔFonKin4*-C, *ΔFonPARP1*, *ΔFonPARP1*-C^E729K^, *ΔFonPARP1*-C, and *ΔFonPARG1* on PDA supplemented with different agents at 7 d. Experiments were independently performed three times with similar results. Data presented in **(B)** represent the means ± SD from three independent experiments, and asterisks indicate significant differences (*p* < 0.05, Student’s *t*-test).

Previous studies have revealed that PARP is engaged in responses to various abiotic stresses in mice and *Arabidopsis* (Shieh et al., 1998; De Block et al., 2005; Vanderauwera et al., 2007; Luo and Kraus, 2012). Therefore, we examined the involvement of *FonPARP1*, *FonPARG1*, and *FonKin4* in the stress responses of *Fon* by comparing the mycelial growth of the deletion mutants, complementation strains, and WT on PDA supplemented with various stress-inducing agents. Overall, *ΔFonPARP1*, *ΔFonPARP1-*C^E729K^, *ΔFonPARG1*, *ΔFonKin4*, and *ΔFonKin4*-C^T462A^ exhibit differential responses to tested stress-inducing agents compared to WT, *ΔFonPARP1-*C, and *ΔFonKin4*-C (Figures 5A,B). In response to cell wall perturbing reagents, *ΔFonPARP1* and *ΔFonPARP1-*C^E729K^ showed increased sensitivity to Congo red (CR) and sodium dodecyl sulfate (SDS) but enhanced tolerance to Calcofluor white (CFW), while *ΔFonPARG1* displayed higher sensitivity to SDS but increased resistance to CR and CFW compared to WT (Figures 5A,B). Concerning oxidative and osmotic stresses, *ΔFonPARP1* and *ΔFonPARP1-*C^E729K^ exhibited higher tolerance to H_2_O_2_, paraquat, and NaCl but became more vulnerable to CaCl_2_ compared to WT (Figures 5A,B). *ΔFonPARG1* was more tolerant to H_2_O_2_ and NaCl but more sensitive to CaCl_2_ (Figures 5A,B). Interestingly, *ΔFonKin4* and *ΔFonKin4*-C^T462A^ exhibited enhanced tolerance to all tested stress-inducing agents (Figures 5A,B). Collectively, these data indicate that FonPARP1, FonPARG1, and FonKin4 have similar functions in oxidative stress responses while playing distinct roles in responses to osmotic and cell wall perturbing stresses.

### 3.8 FonKin4 is a Ser/Thr protein kinase whose enzymatic activity is sufficient for its pathogenicity function

**Figure 6:**
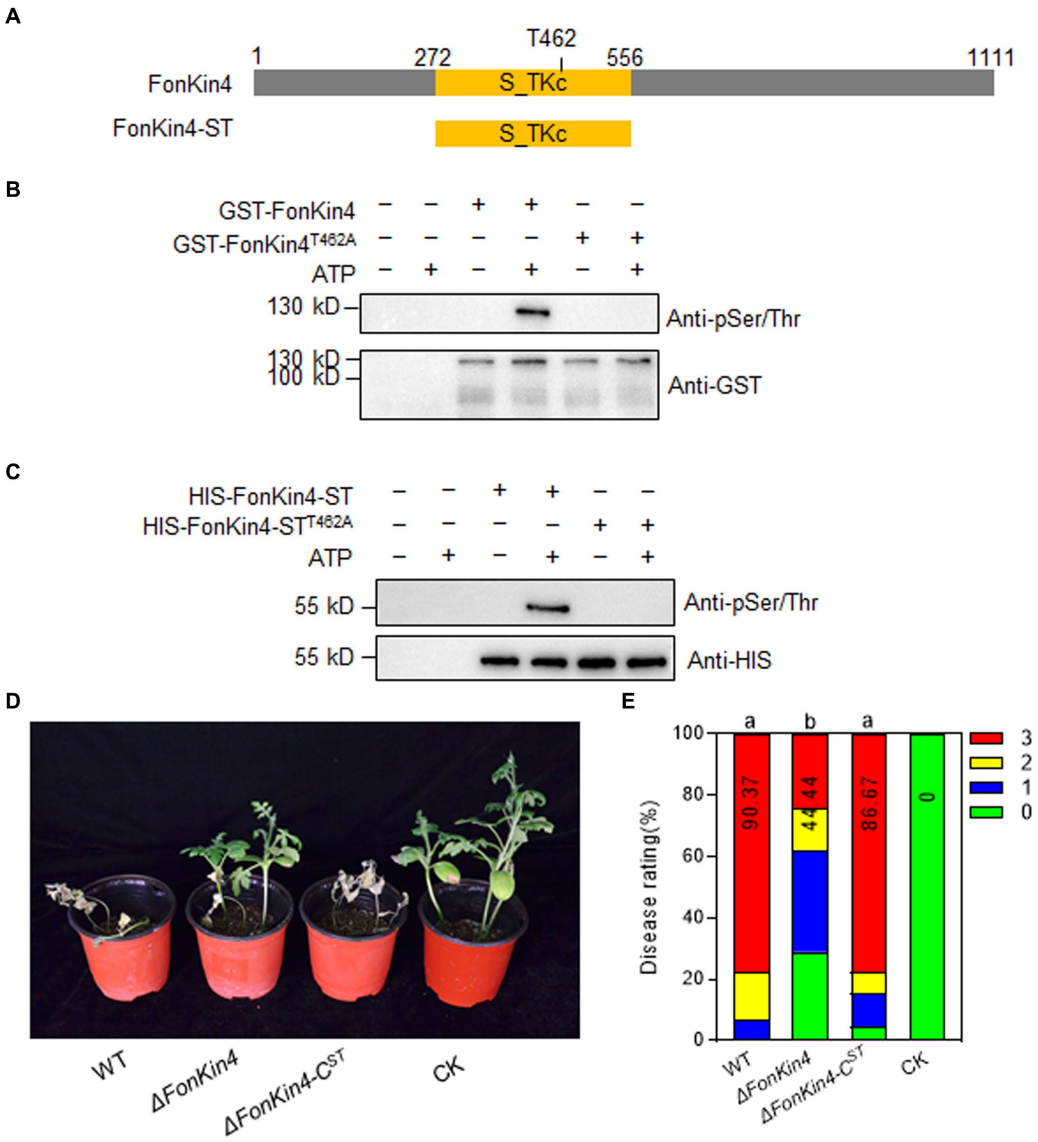
FonKin4 is a Ser/Thr protein kinase and its S_TKc domain is sufficient for its pathogenicity function in *Fon*. **(A)** Schematic representation of the structure of FonKin4 protein. **(B,C)** Intact FonKin4 **(B)** and truncated S_TKc domain-containing fragment FonKin4-ST **(C)** show Ser/Thr protein kinase activity detected by anti-phosphor Ser/Thr antibody. **(D,E)** Disease phenotype **(D)** and disease ratings **(E)** of watermelon plants inoculated with WT, *ΔFonKin4* or *ΔFonKin4*-C^ST^ strains at 21 dpi. Experiments were independently performed three times with similar results. Results from one representative experiment are shown in **(D)**. Data presented in **(E)** represent the means ± SD from three independent experiments, and different letters indicate significant differences from WT (*p* < 0.05, one-way ANOVA).

It was previously shown that ScKin4 possesses protein kinase activity (Pereira and Schiebel, 2005). As FonKin4 contains a typical S_TKc domain (Figure 6A) and shares 53% identity with ScKin4 (Supplementary Figure S7B), we assessed its kinase activity using an *in vitro* phosphorylation assay. The recombinant GST-FonKin4 exhibited self-phosphorylation at Ser/Thr site(s), detected using an anti-phospho Ser/Thr antibody (Figure 6B), confirming FonKin4 as a protein kinase. To explore the importance of the S_TKc domain in FonKin4, we generated a truncated variant FonKin4-ST, containing the S_TKc domain (Figure 6A). We purified the recombinant HIS-FonKin4-ST protein, which also exhibited self-phosphorylation activity in the *in vitro* phosphorylation assay (Figure 6C). To investigate the significance of the specific residue T462 within the S_TKc domain, we mutated the conserved T residue to A in both GST-FonKin4 and HIS-FonKin4-ST. Surprisingly, neither GST-FonKin4^T462A^ nor HIS-FonKin4-ST^T462A^ exhibited self-phosphorylation activity in the *in vitro* phosphorylation assay (Figures 6B,C). These results suggest that FonKin4 is an active Ser/Thr protein kinase, and the S_TKc domain, along with the conserved T462, is essential for FonKin4 kinase activity.

To further assess the role of the FonKin4-ST in the pathogenicity function of FonKin4, we generated a complementation strain *ΔFonKin4-*C^ST^ by introducing a native promoter-driven FonKin4-ST construct into *ΔFonKin4*. Pathogenicity tests revealed that the disease symptoms and rating of the *ΔFonKin4-*C^ST^-inoculated plants were comparable to those of WT-inoculated plants (Figures 6D,E). At 21 dpi, 78% of the plants in both the WT- and *ΔFonKin4-*C^ST^-inoculated groups had succumbed to the disease. In contrast, only 24% of the plants in the *ΔFonKin4*-inoculated group were affected (Figure 6E). These results provide evidence that FonKin4-ST restores the pathogenicity defect in *ΔFonKin4*, highlighting the essential role of enzymatic activity in the pathogenicity function of FonKin4 in *Fon*.

### 3.9 FonKin4 Phosphorylates FonPARP1 to enhance its activity

**Figure 7:**
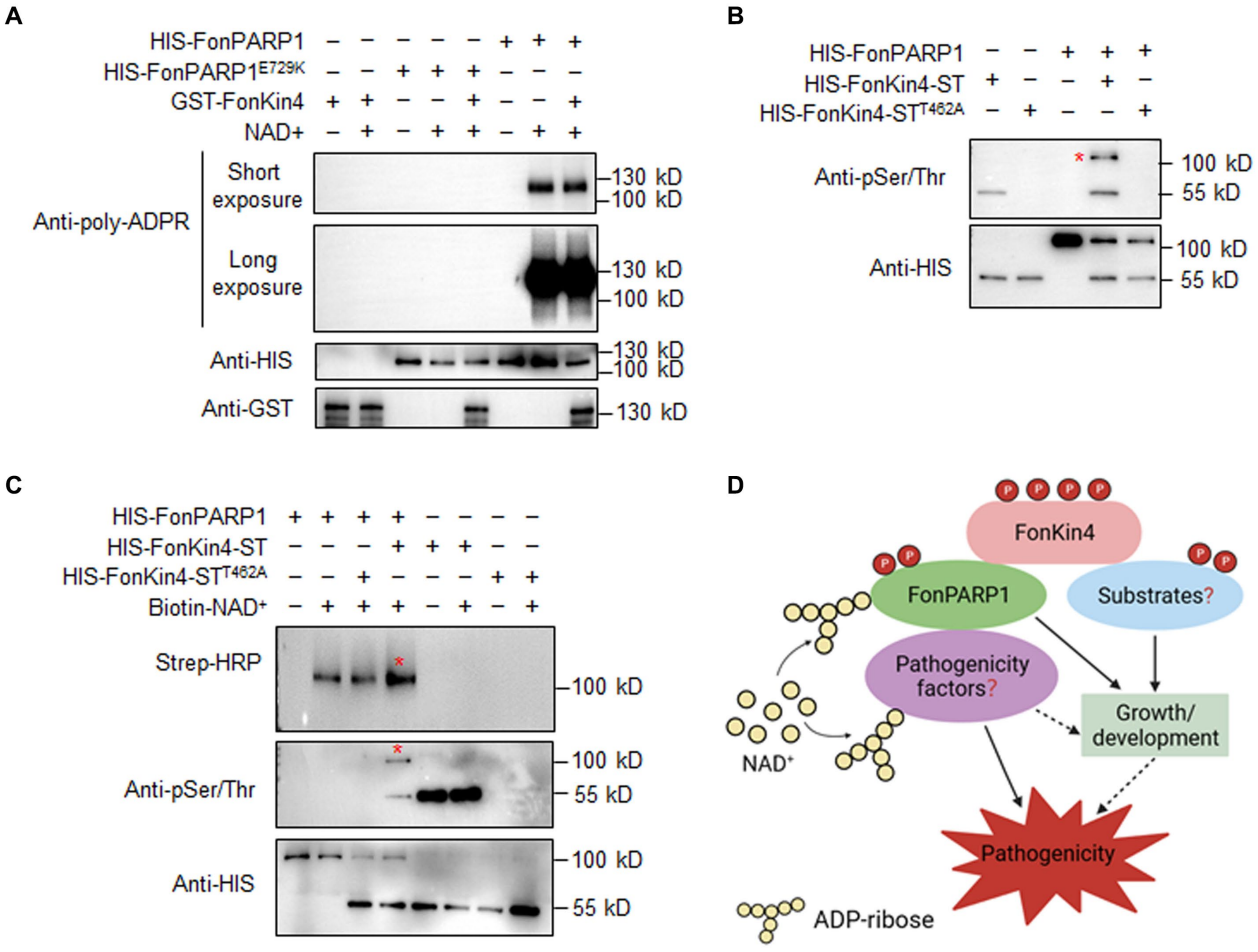
FonKin4 phosphorylates FonPARP1 to enhance its enzymatic activity and a proposed model illustrating the functions of the FonKin4-FonPARP1 cascade in *Fon* pathogenicity. **(A)** FonPARP1 possesses self-PARylation activity but does not PARylate FonKin4 *in vitro*. Short exposure, 1 min; long exposure, 5 min. **(B)** FonKin4 phosphorylates FonPARP1 *in vitro*. **(C)** FonKin4-mediated phosphorylation enhances the self-PARylation activity of FonPARP1 *in vitro*. Experiments were independently performed three times with similar results. Red asterisks in **(B)** and **(C)** indicate the self-PARylated and phosphorylated FonPARP1, respectively. **(D)** A proposed working model deciphering the functions of the FonKin4-FonPARP1 cascade in *Fon* pathogenicity.

The interaction between FonPARP1 and FonKin4 led us to speculate whether FonPARP1 PARylates FonKin4 or FonKin4 phosphorylates FonPARP1. To investigate this, we first examined whether FonPARP1 PARylates FonKin4 *in vitro*. Since GST-FonKin4 is about 130 kD in size, which is close to HIS-FonPARP1, *in vitro* PARylation assays with short and long exposures were conducted to distinguish possible PARylated GST-FonKin4 band from PARylated HIS-FonPARP1 band. No self-PARylated HIS-FonPARP1 band was observed in the absence of NAD^+^ (Figure 7A, *lane 6*). However, when NAD^+^ was added, it resulted in a clear self-PARylated HIS-FonPARP1 band, especially a smear with longer size of the PARylated product in long exposure, indicating that HIS-FonPARP1 was enzymatically active (Figure 7A, *lane 7*). In contrast, no self-PARylated HIS-FonPARP1^E729K^ band was seen in the presence of NAD^+^ (Figure 7A, *lanes 4 and 5*), indicating that mutation of E729 completely abolished the enzymatic activity of FonPARP1. On the other hand, we did not observe any PARylated GST-FonKin4 band, even in long exposure, when GST-FonKin4, HIS-FonPARP1, and NAD^+^ were added to the reaction (Figure 7A, *lane 8*), revealing that FonPARP1 does not PARylate FonKin4 *in vitro*. Next, we explored the possibility of FonKin4-mediated FonPARP1 phosphorylation through *in vitro* phosphorylation assays. In the presence of HIS-FonKin4-ST, we observed a phosphorylated HIS-FonPARP1 band using an anti-phosphor Ser/Thr antibody (Figure 7B, *lane 4*). However, no visible band was detected in the reaction with HIS-FonKin4-ST^T462A^ (Figure 7B, *lane 5*), indicating the function of FonKin4 in phosphorylating FonPARP1 at Ser/Thr site(s).

To determine the functional significance of FonKin4-mediated phosphorylation of FonPARP1, we assayed the enzymatic activity of FonPARP1 by examining its self-PARylation level after being phosphorylated by FonKin4. In the presence of biotin-NAD^+^, the self-PARylation of HIS-FonPARP1 was clearly detected by streptavidin-HRP (Figure 7C, *lane 2 in upper panel*), which was also distinguishable in the presence of HIS-FonKin4-ST^T462A^ (Figure 7C, *lanes 3 in upper panel*). However, when HIS-FonKin4-ST was added to the reaction, we observed a phosphorylated band of HIS-FonPARP1 (Figure 7C, *lane 4 in middle panel*) and enhanced self-PARylation of HIS-FonPARP1 (Figure 7C, *lane 4 in upper panel*) compared to the reactions without HIS-FonKin4-ST or with HIS-FonKin4-ST^T462A^ (Figure 7C, *lanes 2 and 3 in upper panel*). These data indicate that FonKin4-mediated phosphorylation facilitates the activity of FonPARP1.

## 4 Discussion

Protein PARylation, catalyzed by PARPs and mainly degraded by PARGs, has been implicated in various biological processes in mammals and plants (Gibson and Kraus, 2012; Feng et al., 2016; Gibson et al., 2016; Liu et al., 2017; Alemasova and Lavrik, 2019; Yao et al., 2021). However, the functions of PARPs and protein PARylation in plant pathogenic fungi remain elusive. In this study, we revealed that FonPARP1 is required for growth, pathogenicity, and stress responses in *Fon*. Additionally, the protein kinase FonKin4 was characterized to phosphorylate FonPARP1, enhancing its enzymatic activity and playing important roles in growth/development, stress responses, and pathogenicity of *Fon*. Our findings establish a FonKin4-FonPARP1 phosphorylation cascade contributing to *Fon* pathogenicity and demonstrate the importance of PARP1-catalyzed protein PARylation in regulating pathogenicity in both *Fon* and other plant pathogenic fungi.

PARPs are known to be involved in cellular stress signaling related to growth/development, disease occurrence, and immunity in mammals and plants (Luo and Kraus, 2012; Rissel and Peiter, 2019). In our study, *ΔFonPARP1* exhibited slower growth on MM but displayed no alterations in conidiation, macroconidial germination, and morphology (Figure 4). Similarly, knockdown mutant of *AnPrpA* showed significantly reduced growth rates (Semighini et al., 2006). Notably, in *Arabidopsis*, single, double, and triple mutants of *AtPARPs* did not exhibit obvious growth defects (Feng et al., 2015; Rissel et al., 2017). However, AtPARP1, AtPARP2, and AtPARP3 have been implicated in regulating seed germination and root development (Pham et al., 2015; Liu et al., 2017). Therefore, the involvement of FonPARP1 in other growth and developmental processes of *Fon* cannot be ruled out and warrants further investigation. Importantly, we found that the deletion of *FonPARP1* resulted in a significant decline in fungal pathogenicity on watermelon plants (Figures 1A–C), underscoring the requirement of *FonPARP1* for *Fon* pathogenicity. This reduction in pathogenicity in the *ΔFonPARP1* mutant primarily stemmed from a defect in invasive growth within watermelon plants (Figures 1D–G) rather than an impairment in its ability to penetrate the host (Supplementary Figure S6). This pattern mirrors the functions of previously identified pathogenicity factors, including FonNst2, FonPAT2, and FonPUF1, whose deletion mutants exhibited reduced pathogenicity due to compromised *in planta* invasive growth and colonization within host plants (Gao et al., 2022, 2023; Xiong et al., 2023). Our study also revealed that FonPARP1 undergoes self-PARylation *in vitro* (Figure 7A), indicating its active PARP activity in *Fon*. The conserved E988 residue, critical for its PARP activity (Altmeyer et al., 2009; Chen et al., 2019), is essential for FonPARP1 function in *Fon* pathogenicity, as the enzymatically inactive variant FonPARP1^E729K^ (Figure 7A) failed to restore the pathogenicity deficit in the *ΔFonPARP1* mutant (Figures 1A–C). Thus, it is plausible that FonPARP1 PARylates downstream pathogenicity-related factors to regulate *Fon* pathogenicity. Furthermore, FonPARP1 displayed regulatory function in multiple abiotic stress responses, including cell wall-perturbing, osmotic and oxidative stresses (Figure 5), which aligns with earlier observations in mammal (Luo and Kraus, 2012), *Arabidopsis*, and oilseed rape (De Block et al., 2005; Vanderauwera et al., 2007). Collectively, our findings underscore the pivotal roles of FonPARP1 in growth, pathogenicity, and abiotic stress responses in *Fon*.

In mice and *Drosophila melanogaster*, PARGs play essential roles in survival and stress responses through pADPR metabolism (Cortes et al., 2004; Hanai et al., 2004; Koh et al., 2004). *Arabidopsis* plants also employ AtPARG1 and AtPARG2 to modulate immunity against different pathogens and abiotic stresses, including drought, osmotic and oxidative stresses (Adams-Phillips et al., 2010; Li et al., 2011; Feng et al., 2015). Intriguingly, both *atparg1* and *atparg2* mutants did not show noticeable defects in growth, flowering, and seed setting (Feng et al., 2015). Notably, our study found that the deletion of *FonPARG1* did not lead to any discernible deficiencies in vegetative growth, asexual reproduction, and pathogenicity in *Fon* (Figure 4; Supplementary Figure S5). This aligns with previous observations in *F. oxysporum* f. sp. *lycopersici*, where the deletion of *FolPARG1* had no discernible impact on pathogenicity or mycelial growth (Araiza-Cervantes et al., 2018). However, the *ΔFonPARG1* mutant did exhibit altered responses to cell wall-perturbing agents and abiotic stressors (Figure 5), implying the involvement of FonPARG1 in abiotic stress responses. This finding is in line with an earlier observation that the *ΔFolPARG1* mutant showed compromised DNA integrity in *F. oxysporum* f. sp. *lycopersici* (Araiza-Cervantes et al., 2018). Together, these results indicate that FonPARG1 is not directly implicated in vegetative growth, asexual reproduction, or pathogenicity but participates in abiotic stress responses in *Fon*. However, considering that *Fon* possesses a single PARG, further investigations are warranted to elucidate the underlying mechanism of PARG in *Fon* growth and development. Comparative examinations involving the *ΔFonPARG1* mutant may provide additional insights into the functions of FonPARG1 in other growth and developmental processes in *Fon*.

Both IP-LC–MS/MS analysis and protein–protein interaction assays confirmed the association between FonKin4 and FonPARP1 (Figures 2A–C). FonKin4 exhibits a consistent localization pattern, primarily at the septa of mycelia and conidia in *Fon* (Figure 2D), akin to AnKfsA, which localizes at the septa in the conidiophore in *Aspergillus nidulans* (Takeshita et al., 2007). Notably, we observed the subcellular localization of FonPARP1 using the FonPARP1-GFP overexpressing strain and found that FonPARP1 was predominantly localized in the nucleus in another study with its downstream substrates (Wang et al., *unpublished data*). It has been shown that human PARP1 is translocated from nucleus to cytoplasm in response to viral infection (Wang et al., 2022). It is thus likely that FonPARP1 might be translocated from the nucleus to the cytoplasm, where it is phosphorylated by FonKin4, resulting in enhanced PARP activity to promote *Fon* pathogenicity. Different localization of PARPs and their interacting proteins was previously observed in *Arabidopsis*; for instance, AtPARP2 is mainly localized in the nucleus (Feng et al., 2015), whereas AtPDI1, a substrate of AtPARP2 (Yao et al., 2021), is targeted in the endoplasmic reticulum (Zhang et al., 2018). FonKin4 possesses a typical S_TKc domain (Figure 6A) and demonstrates Ser/Thr protein kinase activity, as evidenced by its self-phosphorylation capacity (Figure 6B). This phenomenon is analogous to observations in *S. cerevisiae* that ScKin4 displays kinase activity capable of phosphorylating itself (D’Aquino et al., 2005). In protein kinases, the kinase domains and the conserved residues, like Ser, Thr, or Tyr, in the activation loop are fundamental and typically correlated with their enzymatic activity (Hanks and Hunter, 1995; Krupa et al., 2004). Here, FonKin4-ST, harboring the S_TKc domain, retained its Ser/Thr kinase activity both on itself or its substrate FonPARP1, and restored the pathogenicity defects in the *ΔFonKin4* mutant (Figures 6C–E, 7B,C). Conversely, mutation of T462 in the activation loop completely abolished its Ser/Thr kinase activity (Figures 6B,C, 7B,C). This particular biochemical feature aligns with reports indicating that T209 within the activation loop serves as an essential site for the kinase activity of Kin4 in *S. cerevisiae* (Caydasi et al., 2010; Bertazzi et al., 2011). In this study, FonKin4 further displayed regulatory functions in multiple growth and developmental processes in *Fon*, including vegetative growth, asexual reproduction, and macroconidial morphology (Figure 4), aligning with previous studies suggesting that the ScKin4 serves as a spindle position checkpoint in *S. cerevisiae*, preventing mitotic exit (D’Aquino et al., 2005), and *AnKfsA* deletion resulted in decreased conidiospore production in *A. nidulans* (Takeshita et al., 2007). Importantly, the deletion of *FonKin4* significantly reduced *Fon* pathogenicity on watermelon plants, leading to less severe disease symptoms and a reduced disease rating (Figures 3A–C). Notably, the *ΔFonKin4* mutant exhibited defects in *in planta* invasive growth within watermelon plants (Figures 3D–G), while maintaining its ability to penetrate the plant tissues (Supplementary Figure S6). Thus, FonKin4 likely influences *Fon* pathogenicity by regulating its invasive growth within host plants, consistent with the observed deficiencies in vegetative growth and asexual reproduction (Figure 4). This mechanism parallels findings for FonPUF1, FonNST2, and FonPAT2 in *Fon* pathogenicity (Gao et al., 2022, 2023; Xiong et al., 2023). Furthermore, *FonKin4* participated in stress responses against multiple cell wall-perturbing agents and oxidative stress-inducing factors (Figure 5). Collectively, our findings underscore the multifaceted roles of FonKin4 in diverse biological processes, including growth, development, stress responses, and pathogenicity in *Fon*.

Extensive studies in mammals have unveiled the tight regulation of PARP1 enzymatic activity through distinct mechanisms, encompassing protein interactions and post-translational protein modifications (Fischer et al., 2014; Gibbs-Seymour et al., 2016; Alemasova and Lavrik, 2019). Post-translational modifications, including methylation, phosphorylation, and acetylation are involved in modulating PARP1 activity, among which phosphorylation-based regulatory mechanism has been extensively investigated in human PARP1 (Gagné et al., 2009). For instance, several kinases including JUN N-terminal kinase (JNK), extracellular signal-regulated kinase (ERK), cyclin-dependent kinase 2 (CDK2), checkpoint kinase 2 (CHK2), and AMP-activated protein kinase, directly phosphorylate PARP1 (Kauppinen et al., 2006; Zhang et al., 2007; Wright et al., 2012; Shang et al., 2016; Hsu et al., 2019). In this study, we demonstrated that FonKin4 interacts with and phosphorylates FonPARP1 (Figures 2A–C, 7B,C), which is consistent with an earlier finding that ScKin4 phosphorylates ScBfa1 (Caydasi et al., 2014). Importantly, FonPARP1 did not PARylate FonKin4 *in vitro* (Figure 7A), indicating that FonKin4 unidirectionally phosphorylates FonPARP1. Furthermore, the FonKin4-mediated phosphorylation enhances the self-PARylation activity of FonPARP1 (Figure 7C), suggesting that FonKin4 promotes FonPARP1 activity. This phenomenon mirrors observations in human PARP1, whose activity can be enhanced by protein kinases such as JNK, ERK, CDK2, and CHK2 through direct phosphorylation (Kauppinen et al., 2006; Zhang et al., 2007; Wright et al., 2012; Hsu et al., 2019). In light of the similar functions of FonKin4 and FonPARP1 in the *Fon* pathogenicity (Figures 1, 3), it is plausible that the FonKin4-mediated unidirectional phosphorylation of FonPARP1 forms a FonKin4-FonPARP1 phosphorylation cascade that contributes to *Fon* pathogenicity. Furthermore, both *ΔFonKin4* and *ΔFonPARP1* mutants exhibited comparable enhanced tolerance to exogenous abiotic stresses, including CFW, NaCl, H_2_O_2_, and paraquat (Figure 5), suggesting a potential connection between FonPARP1 and FonKin4 in regulating cell wall, osmotic and oxidative stress responses in *Fon*. Previous research has demonstrated that mammal CDK2 phosphorylates PARP1 at S785 and S786 to promote its activity by creating an increased accessible NAD^+^-binding pocket in the catalytic domain (Wright et al., 2012). Thus, the characterization of FonKin4-catalyzed phosphorylation sites could shed light on the regulatory mechanism of FonPARP1 activity, despite the challenge posed by the relatively large number of Ser (66) and Thr (42) residues in FonPARP1.

## 5 Conclusion

In this study, we delved into the functions of FonPARP1 and FonPARG1, enzymes involved in protein PARylation, in *Fon* pathogenicity. Our investigation unveiled that FonPARP1 assumes a pivotal role in *Fon* pathogenicity, primarily by regulating invasive growth within watermelon plants. It is also implicated in growth and abiotic stress responses. Furthermore, we elucidated that the protein kinase FonKin4 phosphorylates FonPARP1, enhancing its enzymatic activity, and is indispensable for regulating various aspects of *Fon* biology, including vegetative growth, asexual reproduction, stress responses, and ultimately pathogenicity. Based on these findings, we put forth a working model outlining the multifaceted functions of the FonKin4-FonPARP1 cascade in *Fon* (Figure 7D). In this model, FonKin4 phosphorylates FonPARP1, augmenting its enzymatic activity, which, in turn, triggers the PARylation of downstream pathogenicity-related factors, ultimately contributing to *Fon* pathogenicity. Additionally, FonKin4 may exert other functions to indirectly influence various growth and developmental processes in *Fon*. Future investigations will be focused on characterizing the FonPARP1-PARylated pathogenicity factors, further elucidating the molecular network that governs pathogenicity through the FonKin4-FonPARP1 cascade, and underscoring the significance of protein PARylation in plant pathogenic fungi.

## Data availability statement

The original contributions presented in the study are included in the article/Supplementary material, further inquiries can be directed to the corresponding author.

## Author contributions

JW: Conceptualization, Data curation, Formal analysis, Investigation, Methodology, Writing – original draft. YG: Formal analysis, Investigation, Methodology, Writing – original draft. XX: Formal analysis, Investigation, Methodology, Writing – original draft. YY: Formal analysis, Investigation, Writing – original draft. JL: Investigation, Writing – original draft. MN: Writing – review & editing. DL: Conceptualization, Methodology, Writing – review & editing. FS: Conceptualization, Funding acquisition, Supervision, Writing – original draft, Writing – review & editing.
