# Supplementary material for: The Ser/Thr protein kinase FonKin4-poly(ADP-ribose) polymerase FonPARP1 phosphorylation cascade is required for the pathogenicity of watermelon fusarium wilt fungus Fusarium oxysporum f. sp. niveum
Source: Front Microbiol. 2024 Apr 16;15:1397688. doi: 10.3389/fmicb.2024.1397688 (PMC11058995; doi:10.3389/fmicb.2024.1397688)
Supplement: Supplementary file 1 [file Data_Sheet_1.docx]

Supplementary Material

# Supplementary Figures and Tables

## Supplementary Figures


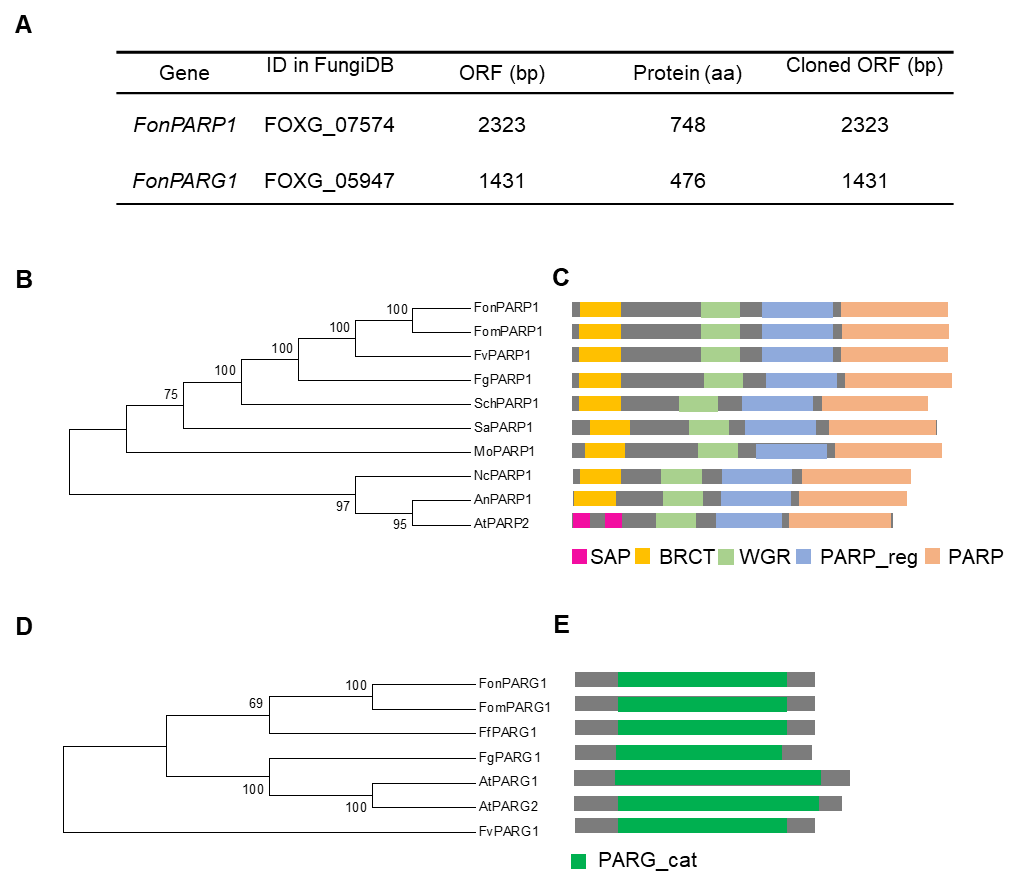


**Supplementary Figure 1.** **Identification of *FonPARP1* and *FonPARG1* in *Fusarium oxysporum* f. sp. *niveum*.** (**A**) Characteristics of *FonPARP1* and *FonPARG1* genes and proteins in *Fon*. (**B**, **C**, **D,** and **E**) Phylogenetic trees (**B** and **D**) and conserved domains (**C** and **E**) of FonPARP1 and FonPARG1 with their homologous from different fungi, including *Fusarium oxysporum* f. sp. *niveum* (Fon), *Fusarium oxysporum* f. sp. *melonis* (Fom), *Fusarium verticillioides* (Fv), *Fusarium graminearum* (Fg), *Magnaporthe oryzae* (Mo), *Neurospora crassa* (Nc), *Aspergillus nidulans* (An), and *Arabidopsis thaliana* (At). The neighbor-joining method was used via MEGA7 software to construct the phylogenic trees. SMART protein database (http://smart.embl-heidelberg.de/) was utilized to analyze protein conserved domains.


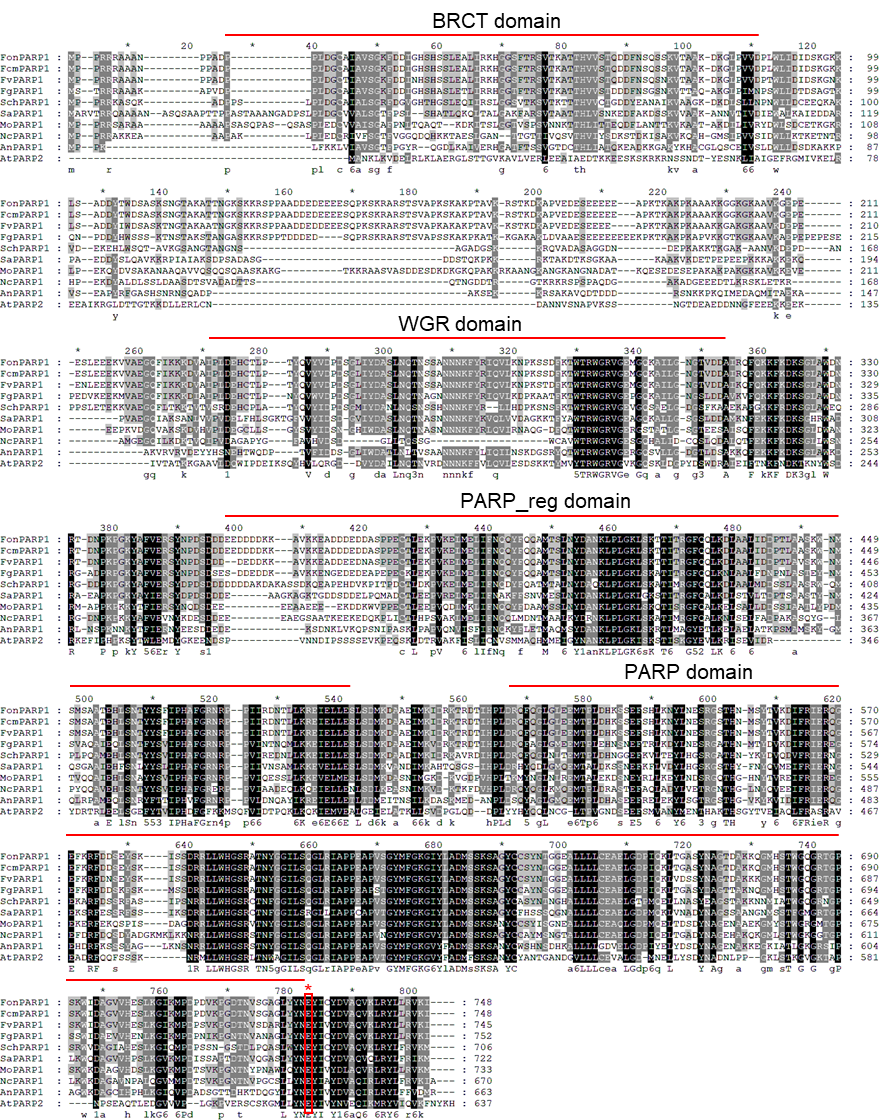


**Supplementary Figure 2.** **Sequence alignment of FonPARP1 with its homologous from different fungal species.** CLUSTALX and GENEDOC programs were used to align amino acid sequences of FonPARP1 and its homologous from other organisms. The conserved domains are highlighted with red lines. The conserved active site is highlighted with a red asterisk.


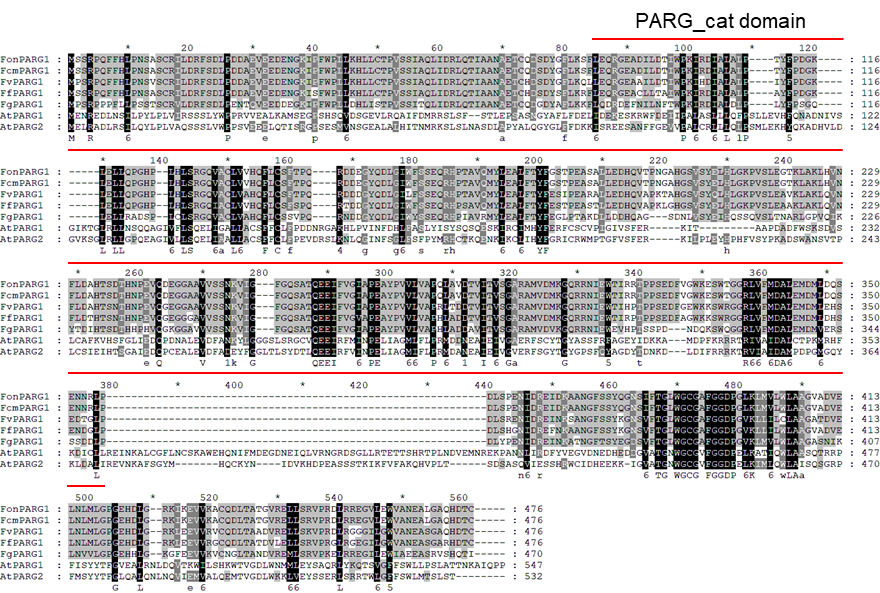


**Supplementary Figure 3.** **Sequence alignment of FonPARG1 with its homologous from different fungal species.** CLUSTALX and GENEDOC programs were used to align amino acid sequences of FonPARG1 and its homologous from other fungal species. PARG_catalytic domain is highlighted with red lines.


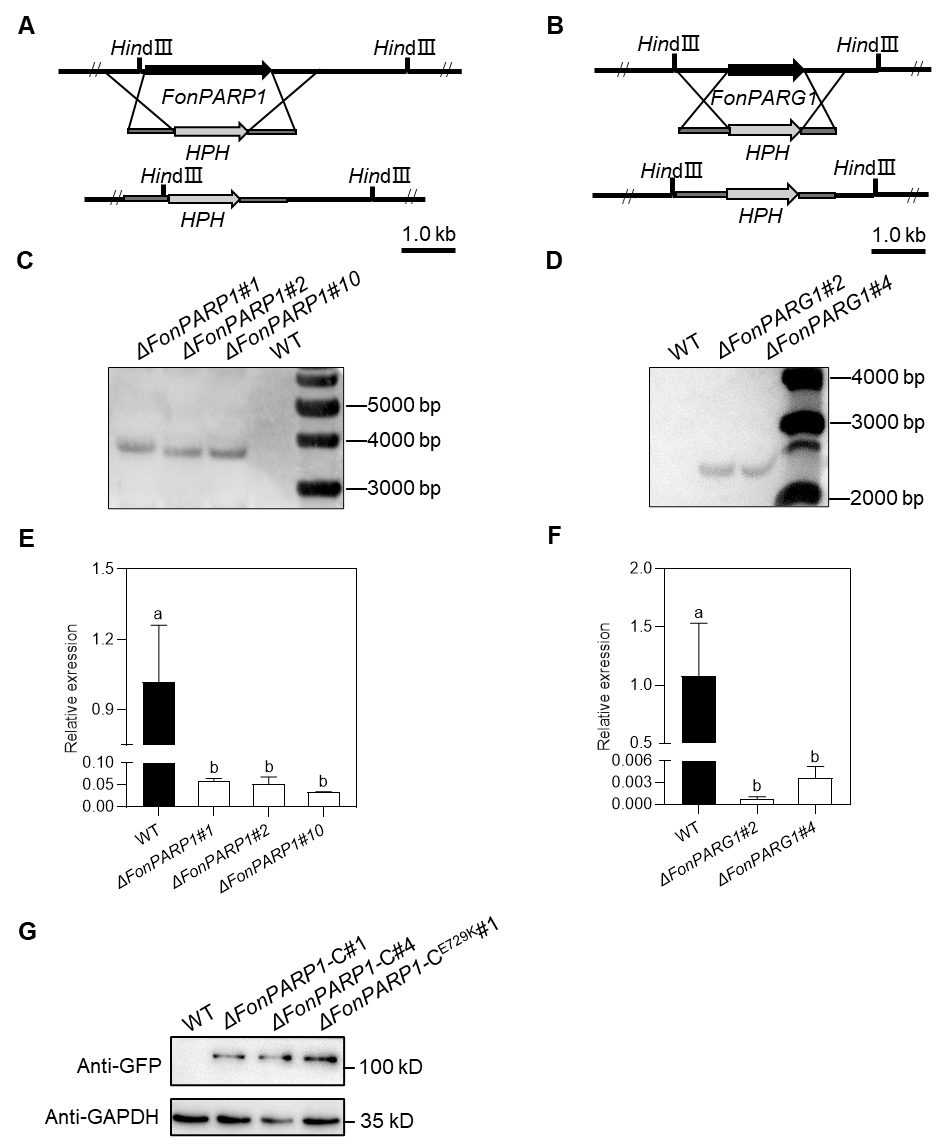


**Supplementary Figure 4.** **Generation and confirmation of *FonPARP1* and *FonPARG1* deletion mutants.** (**A** and **B**) Schematic diagrams showing the strategies to generate *FonPARP1* and *FonPARG1* deletion mutants. *HPH*, hygromycin B resistance gene cassette. (**C** and **D**) Southern blotting of the *FonPARP1* and *FonPARG1* deletion mutants. (**E** and **F**) Relative transcript levels of *FonPARP1* and *FonPARG1* in WT and the corresponding deletion mutants. *FonActin* was used as an internal control. Data are present as the mean ± SD from three independent experiments. Different letters indicate significant differences (*p* < 0.05, one-way ANOVA). (**G**) SDS-PAGE detection of FonPARP1-GFP and FonPARP1^E729K^-GFP protein in WT, *ΔFonPARP1*-C, and *ΔFonPARP1*-C^E729K^ strains.


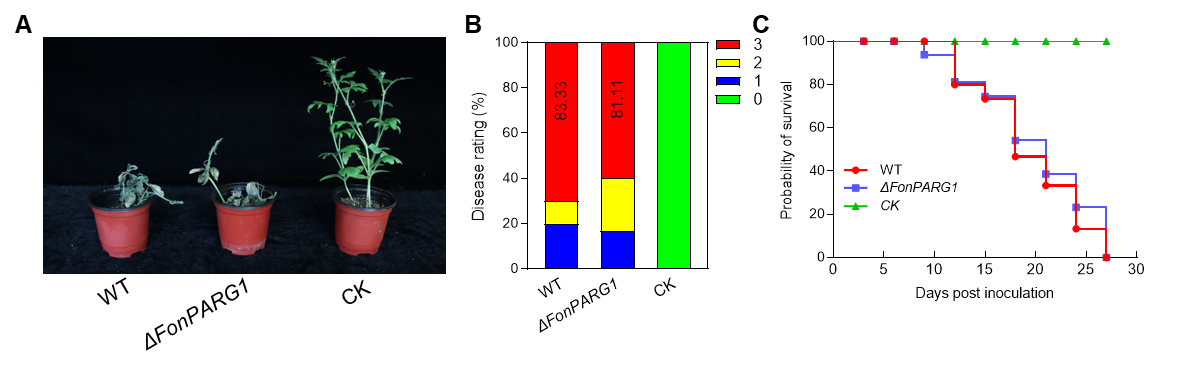


**Supplementary Figure 5.** **FonPARG1 is not involved in *Fon* pathogenicity.** (**A** and **B**) Disease phenotype (**A**) and disease ratings (**B**) of the watermelon plants inoculated with WT or *ΔFonPARG1* strain at 21 dpi. (**C**) Survival curves of the watermelon plants inoculated with WT or *ΔFonPARG1* strains. Experiments were performed independently three times with similar results. Data in (**B**) and (**C**) are present as the averages from three independent experiments.


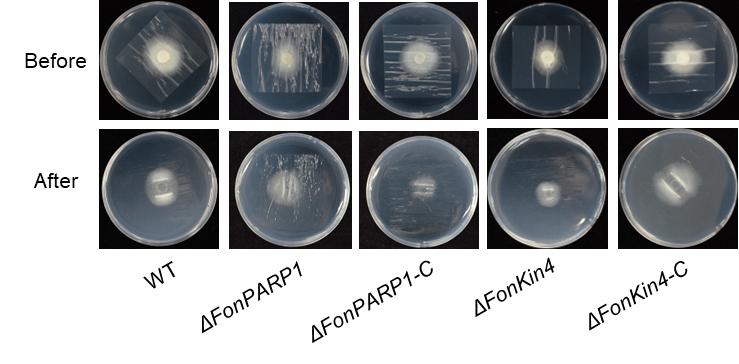


**Supplementary Figure 6.** **Penetration ability of WT, *ΔFonPARP1*, *ΔFonPARP1*-C, *ΔFonKin4*, and *ΔFonKin4*-C strains against cellophane membranes.** Experiments were performed independently three times with similar results.


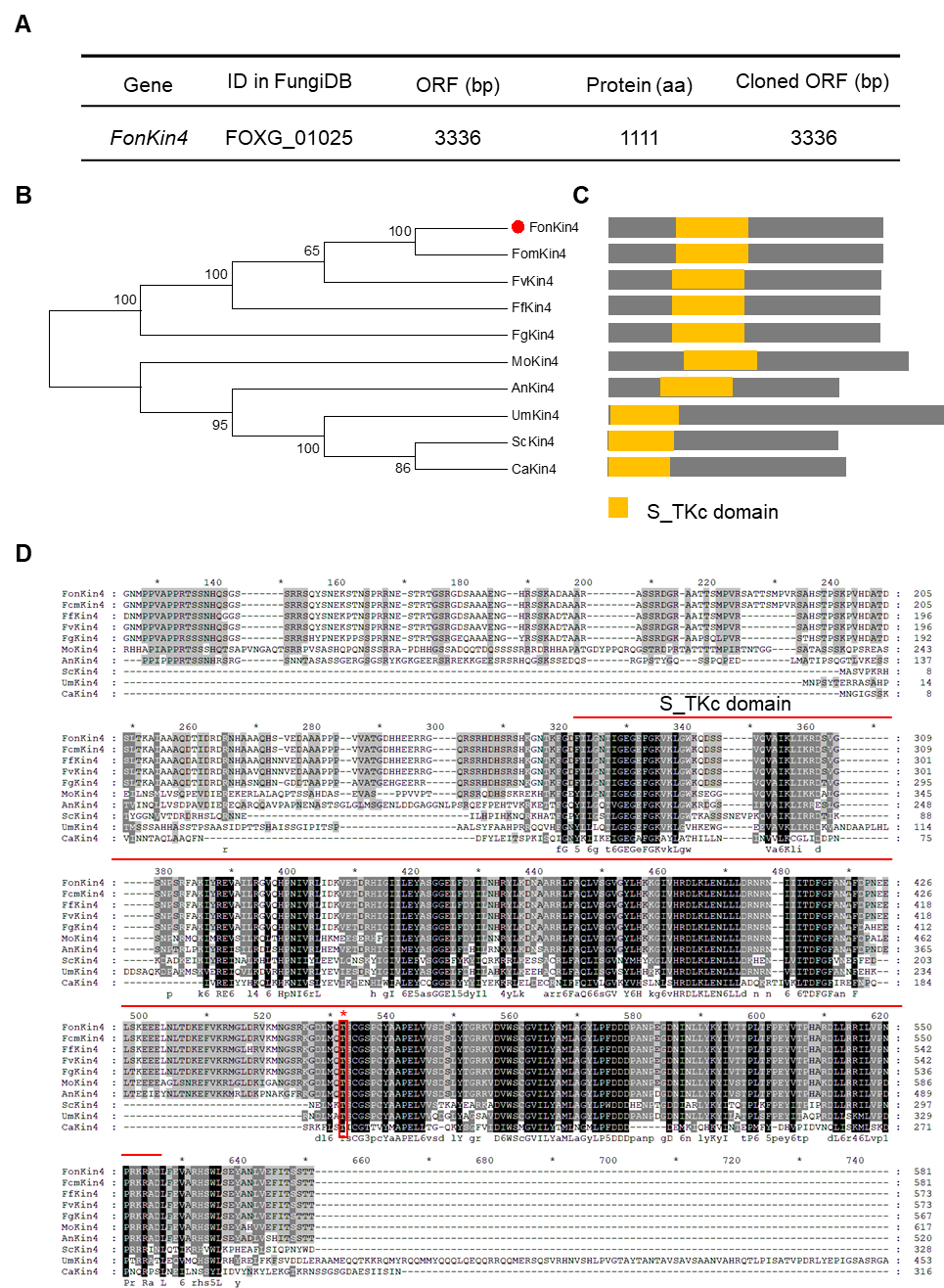


**Supplementary Figure 7.** **Identification of *FonKin4* in *Fon*.** (**A**) Characteristics of *FonKin4* gene and protein in *Fon*. (**B** and **C**) Phylogenetic tree (**B**) and the conserved domains (**C**) of FonKin4 with its homologous from different fungi, including *Fusarium oxysporum* f. sp. *niveum* (Fon), *Fusarium oxysporum* f. sp. *melonis* (Fom), *Fusarium verticillioides* (Fv), *Fusarium graminearum* (Fg), *Magnaporthe oryzae* (Mo), *Aspergillus nidulans* (An), *Ustilago maydis* (Um), *Saccharomyces cerevisiae* (Sc), and *Candida albicans* (Ca). The neighbor-joining method was used via MEGA7 software to construct the phylogenic tree. SMART protein database (http://smart.embl-heidelberg.de/) was utilized to analyze protein conserved domains. (**D**) Sequence alignment of FonKin4 with its homologous from different fungal species. CLUSTALX and GENEDOC programs were used to align amino acid sequences of FonKin4 and its homologous from other fungal species. The conserved S_TKc domain is highlighted with red lines. The conserved active site is highlighted with a red asterisk.


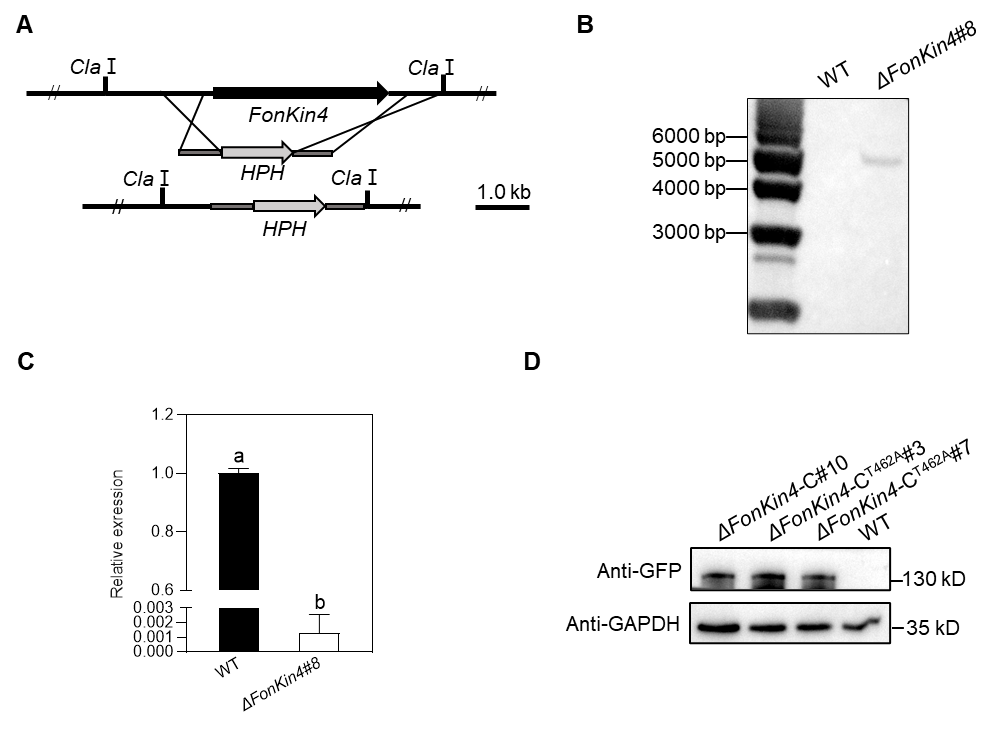


**Supplementary Figure 8.** **Generation and confirmation of *FonKin4* deletion mutant.** (**A**) Schematic diagram showing the strategy to generate *FonKin4* deletion mutant. *HPH*, hygromycin B resistance gene cassette. (**B**) Southern blotting of *FonKin4* deletion mutant. (**C**) Relative transcript level of *FonKin4* in WT and the targeted deletion mutant. *FonActin* was used as an internal control. Data are present as the mean ± SD from three independent experiments. Different letters indicate significant differences (*p* < 0.05, one-way ANOVA). (**D**) SDS-PAGE detection of FonKin4-GFP and FonKin4^T462A^-GFP protein in WT, *ΔFonKin4*-C, and *ΔFonKin4*-C^T462A^ strains.

## Supplementary Tables

**Supplemental Table 1. Primers used in this study.**

| **Primers** | **Sequences (5’-3’)** |
| --- | --- |
| ***Generation of deletion mutants*** | |
| FonPARP1-5’-F | AGAAGTTGCTGTTGTCGTTGCG |
| FonPARP1-5’-R | CAAAATAGGCATTGATGTGTTGACCTCCCAGACCTTGACAATGGATGCC |
| FonPARP1-3’-F | CGTCCGAGGGCAAAGGAATAGAGTAGGACAACGTGACCTTCAGATGCG |
| FonPARP1-3’-R | GACTCCCTCTTGATAATGGCTACTC |
| FonPARP1-YZ-F | ACCCACCCAGGATGAGGTTATT |
| FonPARP1-YZ-R | CGTGAGCGTTCCCGAGATAGTA |
| FonPARP1-E729K-F | GCCTGTACTACAACAAGTACATCTGTTATGATGTCGCCCA |
| FonPARP1-E729K-R | CTTGTTGTAGTACAGGCCGGCACCGGAAACGT |
| FonPARG1-5’-F | CCGAAGGAGTGGTGATACGATG |
| FonPARG1-5’-R | CAAAATAGGCATTGATGTGTTGACCTCCTTTGGGAGGGAAGCGACAGT |
| FonPARG1-3’-F | CGTCCGAGGGCAAAGGAATAGAGTAGGACGAAACCGAAACGGACAGA |
| FonPARG1-3’-R | TCTTCTCAACCTCGCTGCTCTG |
| FonPARG1-YZ-F | TAGCAACATCCTTACTCGCATCTC |
| FonPARG1-YZ-R | TTACCTACGCCAACAACCACTG |
| FonKin4-5’-F | CTTCCCTCCCGTCCACTAAA |
| FonKin4-5’-R | CAAAATAGGCATTGATGTGTTGACCTCCTCGGTCGTATCCGCTTTCTT |
| FonKin4-3’-F | CGTCCGAGGGCAAAGGAATAGAGTAGGGGGATACGAGATTGAGACT |
| FonKin4-3’-R | CACGACGGGATACTATGTTT |
| FonKin4-YZ-F | CCTCCACCTCCAAGTTCCAA |
| FonKin4-YZ-R | TGTTCCCCGTCTACCGCTTA |
| HPH-F | GGAGGTCAACACATCAATGCCTATT |
| HPH-R | CTACTCTATTCCTTTGCCCT |
| ***RT-qPCR*** | |
| FonPARP1-RT-F | AGAGCAGCAAAGTCACAGCG |
| FonPARP1-RT-R | GGAAGCAGAGTCCCAGGTAT |
| FonPARG1-RT-F | TCTTTCATCTTCCCAACTCTGC |
| FonPARG1-RT-R | GAGAAGGTGTTTCAATAGCG |
| FonKin4-RT-F | GACCGCTTCTTCATCTTCGC |
| FonKin4-RT-R | AAGGTTCTTGGTCGTTAGGC |
| FonActin-RT-F | GAGGGACCGCTCTCGTCGT |
| FonActin-RT-R | GGAGATCCAGACTGCCGCTCAG |
| FonOpm12-RT-F | CGATTAGCGAAGACATTCACAAGACT |
| FonOpm12-RT-R | ACGGTCAAGAAGATGCAGGGTAAAGGT |
| ClRps10-RT-F | AGGCTCACCCTAAAAGAAGG |
| ClRps10-RT-R | GGTCAACACAAGGATCTTACT |
| ***Y2H assays*** | |
| FonPARP1-N-AD-F | GCCATGGAGGCCAGTGAATTCATGCCTCCTAGAAGAAGAGCT |
| FonPARP1-N-AD-R | CAGCTCGAGCTCGATGGATCCTGCATCGTCTTCATCATCATC |
| FonPARP1-N-BD-F | ATGGCCATGGAGGCCGAATTCATGCCTCCTAGAAGAAGAGCT |
| FonPARP1-N-BD-R | CCGCTGCAGGTCGACGGATCCTGCATCGTCTTCATCATCATC |
| FonKin4-AD-F | GCCATGGAGGCCAGTGAATTCATGTCGTCGGCTGCCTTACAG |
| FonKin4-AD-R | CAGCTCGAGCTCGATGGATCCCCGGTCCTCGCCACCCCTT |
| FonKin4-BD-F | ATGGCCATGGAGGCCGAATTCATGTCGTCGGCTGCCTTACAG |
| FonKin4-BD-R | CCGCTGCAGGTCGACGGATCCCCGGTCCTCGCCACCCCTT |
| ***GST pull-down assays*** | |
| FonPARP1-HIS-F | GCCATGGCTGATATCGGATCCATGCCTCCTAGAAGAAGAGCT |
| FonPARP1-HIS-R | GTGGTGGTGGTGGTGCTCGAGGATCTTAACGCGGAGGAGGTAA |
| FonKin4-GST-F | GATCTGGTTCCGCGTGGATCCATGTCGTCGGCTGCCTTACAG |
| FonKin4-GST-R | GATGCGGCCGCTCGAGTCGACCCGGTCCTCGCCACCCCTT |
| ***Co-IP assays*** | |
| FonPARP1-GFP-F | ACTCACTATAGGGCGAATTGGGTACTCAAATTGGTTAACAGAAGAAAGAGCCGTGTGG |
| FonPARP1-GFP-R | CACCACCCCGGTGAACAGCTCCTCGCCCTTGCTCACGATCTTAACGCGGAGGAGGTAA |
| FonKin4-GFP-F | ACTCACTATAGGGCGAATTGGGTACTCAAATTGGTTTTCCCTCTCCATTGGAAGATCC |
| FonKin4-GFP-R | CACCACCCCGGTGAACAGCTCCTCGCCCTTGCTCACCCGGTCCTCGCCACCCCTT |
| FonKin4-FLAG-F | CTATAGGGCGAATTGGGTACTCAAATTGGTTTTCCCTCTCCATTGGAAGATCC |
| FonKin4-FLAG-R | CTTTATAATCACCGTCATGGTCTTTGTAGTCCCGGTCCTCGCCACCCCTT |
| ***Phosphorylation activity assays*** | |
| FonKin4-ST-F | GCCATGGCTGATATCGGATCCTTTATTCTGGGCAATACTATCGG |
| FonKin4-ST-R | GTGGTGGTGGTGGTGCTCGAGATCCGCTCTCTTACGGGGAT |
| FonKin4-T462A-F | CATGCAGGCAAGTTGTGGTAGTCCTTGTTATGCTGC |
| FonKin4-T462A-R | CACAACTTGCCTGCATGAGGTCACCCTTCCTA |

**Supplemental Table 2. Putative FonPARP1 interactors in *Fon* identified by IP-MS.**

| **Gene ID** | **Description** | **Peptide** | **Score** |
| --- | --- | --- | --- |
| FOXG_04023 | 2,4-dienoyl-CoA reductase | KALAVELAPKG | 29.02 |
| FOXG_02303 | DUF4045 domain protein | KEAKALGGKL | 43.83 |
| FOXG_22028 | ZnF_C2H2 domain protein | RLNAHIDKC | 34.9 |
| FOXG_00021 | GTP-binding protein | KTSAHRTTNKI | 20 |
| FOXG_17884 | HET domain g protein | KDLNGLVPKRS | 21.21 |
| FOXG_00745 | deoxyhypusine synthase | MTSNSDAPPSAATEAVLVKSEEMPADAQKV | 22.53 |
| **FOXG_01025** | **KIN4 protein kinase** | **RSGSIFGGRSKK** | **20.9** |
| FOXG_01628 | vacuolar protein sorting-associated 1 | KASEIVSQVQ | 22.23 |
| FOXG_02968 | ornithine cyclodeaminase | KITVVNRS | 21.55 |
| FOXG_03093 | hypothetical protein | REDLTAHNIAVTSKC | 21.4 |
| FOXG_03305 | Fluconazole resistance protein 1 | RKSTSSSSKS | 21.14 |
| FOXG_03364 | Metal-dependent phosphatase | RAAVARAKPKI | 25.64 |
| FOXG_03668 | mannosyltransferase | KKTYGSVAKE | 27.23 |
| FOXG_04908 | LITAF domain containing protein | RAMTLTRLKS | 22.47 |
| FOXG_05904 | ATPase | RERPSKTYSWKV | 20.08 |
| FOXG_13653 |  |  |  |
| FOXG_07544 | ATP-dependent RNA helicase | KADERMEFSTSKE | 21.32 |
| FOXG_07847 | hypothetical protein | KWNQYLPPSDNKR | 20.63 |
| FOXG_19738 | hypothetical protein | RYTDPATRQ | 21.87 |
| FOXG_22423 |  |  |  |
| FOXG_19286 |  |  |  |
| FOXG_19375 |  |  |  |
| FOXG_09465 | 40S ribosomal protein S3 | KAAQAAQAAQDARV | 57.47 |
| FOXG_10246 | hypothetical protein | RSGDEKVKI | 25.97 |
| FOXG_11087 | DNA mismatch repair protein | RATVIEQVDRKF | 20.04 |
| FOXG_11101 | SOK1 | RLRSNILARL | 24.26 |
| FOXG_11513 | superoxide dismutase | RINSNTTPPTSSNKM | 22.13 |
| FOXG_12909 | prostaglandin-endoperoxide synthase 1 | KLGGIIEMNVKL | 23.48 |
| FOXG_13000 | gsfR2 | RAQTADASSILSKSGHSWKD | 20.58 |
| FOXG_12504 | hsp70-like protein | RKAGLSDETIKT | 25.48 |
| FOXG_14035 |  |  |  |
| FOXG_13887 | P-element somatic inhibitor protein | REIALIGSRD | 25.67 |
| FOXG_15657 | Zn(II)2Cys6 transcription factor | RTGCATCKK | 22.25 |
| FOXG_17564 | integral membrane protein | RIMNPSRI | 27.25 |
| FOXG_18210 | molybdenum cofactor synthesis 1 | RKGFEAVQKS | 23.44 |
| FOXG_02146 | Splicing coactivator SRm160/300 | RISTQSSVAKS | 22.1 |
| FOXG_02877 | integral membrane protein | KLSGTMSLRI | 26.68 |
| FOXG_03804 | Carboxypeptidase 2 | KNLNINKN | 36.31 |
| FOXG_04031 | hypothetical protein | RDVNRESAVGLFVAGSKV | 23.51 |
| FOXG_05289 | NEDD8-conjugating enzyme | KIWSMKK | 23.58 |
| FOXG_06245 | pathogen-related yeast protein | KVKKPVSKPSTTKE | 24.01 |
| FOXG_07996 | heat shock protein 60 | KLSGGVAVIKV | 24.52 |
| FOXG_09082 | ATPase | KLNIIAGPTGSGKT | 37.68 |
| FOXG_09438 | rnase subunit pop3 | KKVAPIEASWLDVSPDAQYLAPKI | 20.21 |
| FOXG_09509 | SAGA-associated factor 73 | KSHSMGAKRA | 20.22 |
| FOXG_10786 | flavin-binding monooxygenase | RSPTWITSRIGEKF | 23.59 |
| FOXG_10910 | serine/threonine protein kinase | RRTIHFTKN | 27.9 |
| FOXG_20146 |  |  |  |
| FOXG_11520 | NAD-specific glutamate dehydrogenase | RLIKDGKC | 29.13 |
| FOXG_12079 | hypothetical protein | KILNIYKKL | 20.08 |
| FOXG_12811 | Diphosphoinositol polyphosphate phosphohydrolase | KGWVLPKG | 24.26 |
| FOXG_16912 | hypothetical protein | RLPAPLVRR | 21.49 |
| FOXG_17396 | Transcription factor nit-4 | KVFLFTLKR | 21.4 |
